# Investigating the genetic architecture of disease resilience in pigs by genome-wide association studies of complete blood count traits collected from a natural disease challenge model

**DOI:** 10.1186/s12864-021-07835-4

**Published:** 2021-07-13

**Authors:** Xuechun Bai, Tianfu Yang, Austin M. Putz, Zhiquan Wang, Changxi Li, Frédéric Fortin, John C. S. Harding, Michael K. Dyck, PigGen Canada, Jack C. M. Dekkers, Catherine J. Field, Graham S. Plastow

**Affiliations:** 1 Livestock Gentec, Department of Agricultural, Food and Nutritional Science, University of Alberta, Edmonton, AB Canada; 2 Current: ST Genetics, Navasota, TX USA; 3 Department of Animal Science, Iowa State University, Ames, IA USA; 4 Lacombe Research and Development Centre, Agriculture and Agri-Food Canada, Lacombe, AB Canada; 5 Centre de Développement du Porc du Québec, Inc., Quebec City, QC Canada; 6 Department of Large Animal Clinical Sciences, University of Saskatchewan, Saskatoon, SK Canada

**Keywords:** Genome-wide association studies, Disease resilience, Complete blood count, Pigs, Natural disease challenge model

## Abstract

**Background:**

Genetic improvement for disease resilience is anticipated to be a practical method to improve efficiency and profitability of the pig industry, as resilient pigs maintain a relatively undepressed level of performance in the face of infection. However, multiple biological functions are known to be involved in disease resilience and this complexity means that the genetic architecture of disease resilience remains largely unknown. Here, we conducted genome-wide association studies (GWAS) of 465,910 autosomal SNPs for complete blood count (CBC) traits that are important in an animal’s disease response. The aim was to identify the genetic control of disease resilience.

**Results:**

Univariate and multivariate single-step GWAS were performed on 15 CBC traits measured from the blood samples of 2743 crossbred (Landrace × Yorkshire) barrows drawn at 2-weeks before, and at 2 and 6-weeks after exposure to a polymicrobial infectious challenge. Overall, at a genome-wise false discovery rate of 0.05, five genomic regions located on *Sus scrofa* chromosome (SSC) 2, SSC4, SSC9, SSC10, and SSC12, were significantly associated with white blood cell traits in response to the polymicrobial challenge, and nine genomic regions on multiple chromosomes (SSC1, SSC4, SSC5, SSC6, SSC8, SSC9, SSC11, SSC12, SSC17) were significantly associated with red blood cell and platelet traits collected before and after exposure to the challenge. By functional enrichment analyses using Ingenuity Pathway Analysis (IPA) and literature review of previous CBC studies, candidate genes located nearby significant single-nucleotide polymorphisms were found to be involved in immune response, hematopoiesis, red blood cell morphology, and platelet aggregation.

**Conclusions:**

This study helps to improve our understanding of the genetic basis of CBC traits collected before and after exposure to a polymicrobial infectious challenge and provides a step forward to improve disease resilience.

**Supplementary Information:**

The online version contains supplementary material available at 10.1186/s12864-021-07835-4.

## Background

The prevalence of infectious diseases caused by a multitude of pathogens results in high economic losses for the pig industry [1, 2]. Genetic improvement for disease resilience is a practical option to help address the problem of infectious disease as it can ensure production efficiency, because resilient animals are defined as maintaining a relatively undepressed performance in the face of disturbances caused by infection [3, 4]. Disease resilience is a complex trait composed of multiple biological functions, such as growth, health, nutrient status, and other dynamic elements, including the efficiency of immune response and the rate of recovery from infection [5]. This complexity makes disease resilience hard to properly characterize and little is known about the genetic architecture that drives disease resilience. Alternatively, indirect selection of disease resilience based on immune-related traits may be a feasible breeding strategy, because the disease response of an animal largely depends upon its immunity [6, 7].

Blood cells comprise white blood cells, red blood cells, and platelets that are important elements of an animal’s immune status [8]. Complete blood count (CBC) is one of the most common clinical tests performed to evaluate concentrations and relative proportions of these circulating blood cells, which may help to uncover the layers of immune system complexity [9]. Our previous study [10] found that CBC traits collected from blood samples of pigs in both healthy and challenged conditions at 2-weeks before, and 2 and 6-weeks after exposure to a polymicrobial challenge were moderately to highly heritable (0.08 ± 0.04 to 0.53 ± 0.05). Changes of each CBC trait between blood samples collected at different time points (e.g. the change of a CBC level from 2-weeks before to 2-weeks after exposure to the challenge) were also found to be heritable, with estimates ranging from 0.06 ± 0.04 to 0.24 ± 0.04 [10]. These heritability estimates indicate the importance of the genetic component of CBC traits. Moreover, significant genetic correlations (either positive or negative) were found for several CBC traits collected after exposure to the challenge with the economically important production traits of grow-to-finish growth rate (GFGR) and treatment rate (TR) in response to the polymicrobial challenge (− 0.82 ± 0.47 to 0.89 ± 0.26) [10], which may further indicate the potential of developing those CBC traits as indicator traits of disease resilience. In addition to these significant genetic correlations for CBC with GFGR and TR, our previous study [10] also found high genetic correlations (≥ 0.40 ± 0.04) between the CBC traits. Changes in CBC traits between each time point were also found to be genetically correlated, with significant estimates ranging from − 0.42 ± 0.21 to − 0.92 ± 0.11 to 0.44 ± 0.22 to 0.98 ± 0.03 [10]. This allows multivariate models to be used for joint analyses of these genetically correlated traits, which provides the potential to improve statistical power and explore pleiotropy [11–14].

To date, some quantitative trait loci (QTL) have been identified for some blood cell traits in pigs under either healthy or disease challenged status by linkage and association analyses [15–22]. However, due to the use of a pathogen-specific challenge or a relatively low density of genetic markers, the genetic components of blood cell traits in pigs under typical commercial environments, where multiple disease-causing pathogens are present, remains largely unknown.

In this study, CBC traits were collected from pigs in a natural polymicrobial disease challenge model, as described by Bai et al. [10]. Standard univariate genome-wide association studies (GWAS) and multivariate GWAS based on a relatively high-density panel of 465,910 autosomal single-nucleotide polymorphisms (SNPs) were conducted for these CBC traits. The objectives were: (1) to reveal the genomic regions associated with the CBC traits and with their changes in response to the polymicrobial challenge; and (2) to explore the underlying genetic architecture for disease resilience of pigs in the face of a polymicrobial infectious challenge.

## Results

### Descriptive statistics and genetic parameters

Descriptive statistics (mean, standard deviation, minimum, maximum, and distribution) for the CBC data after removing the outliers, are shown in Additional file 1: Fig. S1, S2 and S3. Details about genetic parameters for the evaluated CBC traits, including heritabilities and genetic correlations, can be found in our previous study [10], which used the same 2593 genotyped animals. In addition to the genetic correlations with resilience already reported for these data by Bai et al. [10], we also found significant genetic correlations for platelet concentration in Blood 3 collected at 2-weeks after exposure to a polymicrobial infectious challenge with GFGR (0.40 ± 0.22) and TR (− 0.46 ± 0.26), and for the change of monocyte concentration from Blood 1 to Blood 3 (MONOΔ13) collected at 2-weeks before and 2-weeks after exposure to the challenge with GFGR (0.63 ± 0.21).

### Population structure

As false positive results can be introduced in GWAS by confounding effects due to population stratification, multidimensional scaling (MDS) plots (Additional file 2: Fig. S4) were generated to provide a visualization of the population structure in the first three dimensions (C1, C2, and C3). Animals tended to cluster by farm of origin, as they shared a similar genetic background when they came from the same farm. Since batches were nested within farms and coded uniquely, population stratification associated with the farm effect was accounted for in the association analysis model by fitting the fixed effect of batch. The genomic inflation factors of single-step (SSGWAS) for the CBC traits ranged from 0.98 to 1.06, suggesting that there was no population stratification that confounded the GWAS results.

### Association results and estimates for SNP effects

#### White blood cell traits

Five genomic regions were found to be significantly associated with white blood cell traits at a genome-wise false discovery rate (*FDR*) of 0.05 by univariate SSGWAS. Of note, SNPs located on *Sus scrofa* chromosome (SSC) 4, SSC10, and SSC12 were found to be associated with eosinophil concentration in Blood 3, which was collected 2 weeks after exposure to the challenge (EOSB3). Meanwhile, SNPs on SSC2 and two adjacent floating SNPs (significant SNPs without a group of supportive SNPs) on SSC9 were identified to be associated with MONOΔ13. The Manhattan and quantile-quantile (Q-Q) plots for EOSB3 and MONOΔ13 are shown in Additional file 2: Figs. S5 and S6. Top lead SNPs (the most significant SNP with a group of supportive SNPs) for significant associations (genome-wise *FDR < 0.05*) with EOSB3 and MONOΔ13 are shown in Table 1. For EOSB3, the additive genetic variances explained by the 1 Mb window of the top lead SNPs (SNP1, SNP2, SNP3) and their adjacent SNPs on SSC4, SSC10, and SSC12 were estimated to be 0.46, 0.35, and 0.53% of the additive genetic variance for EOSB3, respectively. SNP4 was a floating SNP on SSC2 and its 1 Mb window explained 0.12% of the additive genetic variance for MONOΔ13. The 1 Mb window for SNP5, the top lead SNP on SSC9, was estimated to explain about 1.23% of the additive genetic variance for MONOΔ13.

**Table 1:** Top significant SNPs identified by univariate SSGWAS for significant associations with white blood cell traits at a genome-wise false discovery rate (*FDR*) of 0.05

Trait^a^	Blood^b^	SNP ID	SNP status^c^	SSC^*d*^	SNP position (bp)	MAF^e^	FDR	GVar (%)^f^	1-Mb window start SNP position^g^ (bp)	Dominance effect ± standard error	Additive effect^h^ ± standard error
EOS	Blood 3	SNP1: rs336560074	Top lead	4	93,647,202	0.31	0.006	0.46	93,331,316	0.001 ± 0.01	**−0.05 ± 0.01**^i^
EOS	Blood 3	SNP2: rs346258273	Top lead	10	8,186,695	0.08	0.03	0.35	7,396,201	**−0.09 ± 0.04**	**0.14 ± 0.04**
EOS	Blood 3	SNP3: rs339860061	Top lead	12	36,308,994	0.13	0.003	0.53	35,450,868	0.002 ± 0.02	**−0.06 ± 0.01**
MONO	Δ13	SNP4: rs321357560	Top floating	2	120,341,201	0.47	0.049	0.12	120,219,793	−0.03 ± 0.02	**0.08 ± 0.02**
MONO	Δ13	SNP5: rs327963623	Top lead	9	105,461,701	0.43	0.049	1.23	105,461,701	−0.02 ± 0.02	**−0.08 ± 0.02**

^a^*EOS* eosinophil concentration, *MONO* monocyte concentration

^b^Blood 3: the CBC measures in Blood 3 collected at 2-weeks after the challenge; Δ13: the change of CBC measures from Blood 1 collected at 2-weeks before the challenge to Blood 3 collected at 2-weeks after a polymicrobial infectious challenge

^c^Top lead: the most significant SNP with a group of supportive SNPs; Top floating: the most significant SNP without a group of supportive SNPs

^d^*Sus scrofa* chromosome

^e^Minor allele frequency

^f^The largest percentage of additive genetic variance explained by the top significant SNP and its adjacent SNPs in a 1 Mb window

^g^Positions of the start SNP for the 1 Mb window segment with the largest amount of additive genetic variance

^h^Estimates of additive effects per additional copy of the “B” allele. When the dominance effect was not significant (*p > 0.05*) the estimate of the additive effect was based on a model without the dominance effect

^i^Significant estimates of additive and dominance effects are highlighted in bold (*p < 0.05*)

Estimates of additive and dominance effects for the top significant SNPs (genome-wise *FDR < 0.05*, including both top lead and top floating SNPs) associated with EOSB3 and MONOΔ13 are summarized in Table 1. A significant dominance effect (*p < 0.05*) was only identified for SNP2, which was associated with EOSB3. Estimates of additive effects were found to be significant (*p < 0.05*) for all SNPs that were associated with EOSB3 and MONOΔ13. For EOSB3, estimates of additive effects were − 0.05 ± 0.01, 0.14 ± 0.04, and − 0.06 ± 0.01 for SNP1, 2 and 3, respectively. Estimates of additive effects for MONOΔ13 were 0.08 ± 0.02 and − 0.08 ± 0.02 for SNP 4 and 5, respectively.

Due the relatively low genetic correlations and large standard errors between white blood cell traits [10], no genomic region was found to be significantly associated with white blood cell traits at *FDR < 0.05* from multivariate SSGWAS.

#### Red blood cell and platelet traits

Nine genomic regions were found to be significantly associated with red blood cell and platelet traits at the genome-wise *FDR* of 0.05 by univariate and or multivariate SSGWAS. The Manhattan plots and Q-Q plots are shown in Additional file 2: Figs. S7, S8, S9, S10, S11 and S12. The four top lead SNPs for significant associations (genome-wise *FDR < 0.05*) and estimates of additive genetic variances explained by these top lead SNPs and their adjacent SNPs in a 1 Mb window are summarized in Table 2. Five floating SNPs (genome-wise *FDR < 0.05*) that explained small amounts of the additive genetic variance (0.05 to 0.21%) for associated red blood cell and platelet traits were found and are summarized in Table 3. Of note, several pleiotropic SNPs associated with red blood cell or platelet traits were identified by multivariate SSGWAS of CBC traits in Blood 1, 3, and 4 (collected at 2-weeks before, 2- and 6-weeks after the challenge, respectively). High genetic correlations were found between mean corpuscular hemoglobin (MCH), mean corpuscular volume (MCV), and red blood cell concentration (RBC) traits (Additional file 1: Table S1), and also between all three sampling time points for each of these traits (≥ 0.77 ± 0.08) [10]. Therefore, pleiotropic SNP7 on SSC6 was identified as the top lead and pleiotropic SNP for MCH in Blood 1 and for both MCV and RBC traits in all three blood samples (Table 2). The percentage of additive genetic variance explained by the 1 Mb window of SNP7 and its adjacent SNPs ranged from 0.29 to 0.57% for its associated traits. Moreover, SNP8 was the top lead and pleiotropic SNP on SSC8, which was associated with MCH, MCV, and RBC traits in all three blood samples. The percentages of additive genetic variance explained by SNP8 and its adjacent SNPs in a 1 Mb window were estimated to range from 0.28 to 0.35% for its associated traits. SNP9 on SSC17 was the top lead and pleiotropic SNP for mean platelet volume (MPV) in Blood 1 and 4. Together with adjacent SNPs in a 1 Mb window, SNP7 was estimated to explain about 0.49 and 0.40% of the additive genetic variances for MPV in Blood 1 and 4, respectively. Significant associations (genome-wise *FDR < 0.05*) for SNP7 with MCV in Blood 1 (genome-wise *FDR* = 0.003) and for SNP8 with MCV in Blood 4 (genome-wise *FDR =* 0.04) were also found by univariate SSGWAS but at a lower significance level compared to the multivariate SSGWAS. Meanwhile, univariate SSGWAS only indicated suggestive associations (genome-wise *FDR* of 0.10) for SNP8 with RBC in Blood 1 (genome-wise *FDR = 0.09*) and with MCV in Blood 1 (genome-wise *FDR = 0.08*).

**Table 2:** Top lead SNPs^a^ identified by univariate and multivariate SSGWAS for significant associations with red blood cell and platelet traits at a genome-wise false discovery rate (*FDR*) of 0.05

SNP ID	SSC^b^	SNP position (bp)	MAF^c^	Trait^d^	Blood^e^	FDR	GVar (%)^f^	1-Mb window start SNP^g^ position (bp)	Dominance effect ± standard error	Additive effect^i^ ± standard error
SNP6: rs336055186	4	91,591,493	0.38	MCHC	Blood 3	0.04	1.15	91,291,800	**1.08 ± 0.02**^j^	**−2.13 ± 0.02**
SNP7^h^: rs325274805	6	28,511,423	0.41	MCH	Blood 1	0.04	0.29	28,110,554	0.10 ± 0.07	**−0.25 ± 0.06**
				MCV	Blood 1	0.001	0.57	28,110,554	**0.29 ± 0.07**	**−0.73 ± 0.06**
					Blood 3	0.002	0.49	28,096,004	0.05 ± 0.04	**−0.45 ± 0.04**
					Blood 4	0.002	0.48	28,096,004	**−0.12 ± 0.04**	**−0.53 ± 0.04**
				RBC	Blood 1	0.01	0.44	28,110,554	−0.02 ± 0.07	0.08 ± 0.06
					Blood 3	0.01	0.44	28,110,554	0.001 ± 0.04	**0.08 ± 0.04**
					Blood 4	0.03	0.40	28,110,554	−0.04 ± 0.05	0.06 ± 0.04
SNP8^h^: rs344612650	8	41,156,538	0.45	MCH	Blood 1	0.01	0.36	40,257,441	**0.15 ± 0.06**	**0.20 ± 0.05**
					Blood 3	0.04	0.36	40,257,441	**0.11 ± 0.04**	**0.16 ± 0.04**
					Blood 4	0.04	0.35	40,257,441	−0.006 ± 0.04	**0.18 ± 0.04**
				MCV	Blood 1	0.03	0.33	40,219,864	**0.35 ± 0.06**	**0.59 ± 0.05**
					Blood 3	0.006	0.27	40,219,864	**0.19 ± 0.04**	**0.38 ± 0.04**
					Blood 4	0.002	0.33	40,219,864	0.04 ± 0.04	**0.51 ± 0.04**
				RBC	Blood 1	0.007	0.31	40,219,864	0.01 ± 0.06	−0.08 ± 0.05
					Blood 3	0.02	0.31	40,219,864	−0.02 ± 0.04	−0.06 ± 0.04
					Blood 4	0.03	0.30	40,219,864	0.01 ± 0.04	−0.06 ± 0.04
SNP9^h^: rs323125939	17	59,739,745	0.34	MPV	Blood 1	0.02	0.49	59,053,639	0.002 ± 0.10	**0.28 ± 0.08**
					Blood 4	0.04	0.40	59,053,639	−0.09 ± 0.07	**0.25 ± 0.05**

^a^The most significant SNP with a group of supportive SNPs

^b^*Sus scrofa* chromosome

^c^Minor allele frequency

^d^*MCHC* mean corpuscular hemoglobin concentration, *MCH* mean corpuscular hemoglobin, *MCV* mean corpuscular volume, *RBC* red blood cell concentration, *MPV* mean platelet volume

^e^Blood 1, Blood 3, and Blood 4: CBC measures in blood samples collected at 2-weeks before, and 2- and 6-weeks after a polymicrobial infectious challenge

^f^The largest percentage of additive genetic variance explained by the top lead SNP and its adjacent SNPs in a 1 Mb window

^g^Positions of the start SNP for the 1 Mb window segment with the largest amount of additive genetic variance

^h^SNPs identified and results estimated by multivariate SSGWAS

^i^Estimates of additive effects per additional copy of the “B” allele. When the dominance effect was not significant (*p > 0.05*) the estimate of the additive effect was based on a model without the dominance effect

^j^Significant estimates of additive and dominance effects are highlighted in bold (*p < 0.05*)

**Table 3:** Top floating SNPs^a^ identified by univariate and multivariate SSGWAS for significant associations with red blood cell and platelet traits at a genome-wise false discovery rate (*FDR*) of 0.05

SNP ID	SSC^b^	SNP Position (bp)	MAF^c^	Trait^d^	Blood^e^	FDR	GVar (%)^f^	1-Mb window start SNP^g^ position (bp)	Dominance effect ± standard error	Additive effect^i^ ± standard error
SNP10^h^: rs319452131	1	18,792,764	0.37	MCV	Blood 1	0.003	0.18	18,536,535	−0.16 ± 0.18	**0.52 ± 0.14**^j^
					Blood 3	0.004	0.21	18,546,024	0.03 ± 0.13	**0.52 ± 0.10**
					Blood 4	0.02	0.15	18,536,535	−0.02 ± 0.14	**0.37 ± 0.11**
SNP11^h^: rs1109789977	5	64,520,638	0.31	PLT	Blood 1	0.001	0.09	63,861,170	−3.80 ± 6.83	**26.78 ± 5.18**
					Blood 3	0.001	0.09	63,861,170	−9.28 ± 7.44	**23.92 ± 5.39**
					Blood 4	0.03	0.05	63,861,170	−10.57 ± 7.40	**18.33 ± 5.46**
SNP12^h^: rs320615395	9	40,919,049	0.45	MCH	Blood 1	0.03	0.05	39,919,771	0.07 ± 0.08	**0.21 ± 0.07**
					Blood 3	0.04	0.07	40,490,005	−0.04 ± 0.05	**0.19 ± 0.05**
					Blood 4	0.04	0.06	40,490,005	0.03 ± 0.05	**0.21 ± 0.05**
SNP13: rs80784550	11	13,749,336	0.12	MCHC	Δ14	0.02	0.07	13,011,748	1.89 ± 2.42	2.38 ± 2.33
SNP14^h^: rs323585109	12	22,234,265	0.3	MCV	Blood 3	0.04	0.08	21,749,390	−0.06 ± 0.14	**−0.40 ± 0.10**

^a^The most significant SNP without a group of supportive SNPs

^b^*Sus scrofa* chromosome

^c^Minor allele frequency

^d^*MCV* mean corpuscular volume, *PLT* platelet concentration, *MCH* mean corpuscular hemoglobin, *MCHC* mean corpuscular hemoglobin concentration

^e^Blood 1, Blood 3, and Blood4: CBC measured in blood samples collected at 2-weeks before, and 2- and 6-weeks after a polymicrobial infectious challenge; Δ14: the change of CBC measures from Blood 1 collected at 2-weeks before the challenge to Blood 4 collected at 6-weeks after the challenge

^f^The largest percentage of additive genetic variance explained by the top significant SNP and its adjacent SNPs in a 1 Mb window

^g^Positions of the start SNP for the 1 Mb window segment with the largest amount of additive genetic variance

^h^SNPs identified and results estimated by multivariate SSGWAS

^i^Estimates of additive effects per additional copy of the “B” allele. When the dominance effect was not significant (*p > 0.05*) the estimate of the additive effect was based on a model without the dominance effect

^j^Significant estimates of additive and dominance effects are highlighted in bold (*p < 0.05*)

For red blood cell and platelet traits, the estimates of additive and dominance effects for the top lead SNPs are summarized in Table 2 and for the top floating SNPs in Table 3. Of note, the additive effects for pleiotropic SNPs showed a tendency of affecting each CBC trait in the three blood samples in the same way, including SNP7 for MCV, SNP8 for MCH and MCV, SNP9 for MPV, SNP10 for MCV, SNP11 for PLT, and SNP12 for MCH. For pleiotropic SNP8, no significant additive effect was found for RBC traits.

### Candidate genes and functional enrichment results

Browsing regions for candidate genes located within a maximum distance of 1 Mb on either side of the lead SNPs based on a genome-wise *FDR < 0.1* for the associated CBC traits are summarized in Additional file 3: Table S2. Candidate gene functions for each CBC trait were explored with the Ingenuity Pathway Analysis (IPA) database. The top five enriched (*p < 0.05*) biological functions among diseases, molecular and cellular functions, and physiological system development and function categories for each CBC trait are summarized in Additional file 4. Enriched functions such as inflammatory responses, cell-to-cell signaling and interaction, cellular development, cell morphology, cellular growth and proliferation, and hematological system development and function were commonly identified for the candidate gene lists for white blood cell traits collected after exposure to the challenge, and / or the pleiotropic candidate gene lists for red blood cell and platelet traits collected before and after exposure to the challenge.

Candidate genes that have been reported by previous studies of pigs, human, mice, or rats to be functionally and biologically related to the same category of blood cells, as explored here, are summarized in Table 4. A group of immunity genes on SSC2 has been reported to be functionally and biologically related to monocytes, including *TICAM2 (toll-like receptor adaptor molecule 2)*, *TMED7(transmembrane emp24 domain-containing protein 7 precursor)*, and *CDO1 (cysteine dioxygenase type 1)*, which were located proximal to SNP4, and *COMMD10 (COMM domain containing 10)*, which harbored SNP4 (Table 4). An overview of the location of these candidate genes and the distribution of all the SNPs in this region on SSC2 is shown in the linkage disequilibrium (LD) haplotype map in Additional file 3: Fig. S13. SNP6 on SSC4 is intronic within candidate gene *SPTA1 (spectrin alpha, erythrocytic 1)* and the LD haplotype map for this region is shown in Additional file 3: Fig. S14. In Table 4, a group of candidate genes, including *THAP11 (THAP domain containing protein 11)*, *PSMB10 (proteasome subunit beta type 10)*, *LCAT (lecithin-cholesterol acyltransferase)*, and *SLC12A4 (Potassium/Chloride Cotransporter 1)*, was reported to be functionally related to red blood cells and were located close to SNP7 in the same haplotype block on SSC6 (Additional file 3: Fig. S15). SNP8 on SSC8 was found to be in LD (r^2^ > 0.30) with SNPs in the *PDGFRA (platelet derived growth factor receptor alpha)* gene (Additional file 3: Fig. S16).

**Table 4:** Candidate genes located within 1 Mb on either side of the top significant SNPs^a^ that have been reported by previous studies of pigs, human, mice, and rats to be functionally and biologically related to CBC traits

SNPID	Traits^b^	Browsing region	Candidate genes and locations
SNP1	Eosinophils	SSC4: 92,647,202 bp – 94,647,202 bp	*ARHGEF2* (94,026,391 bp – 94,082,206 bp)
SNP2	Eosinophils	SSC10: 7,186,695 bp – 9,186,695 bp	*TGFB2* (8,327,779 bp – 8,435,306 bp)
SNP3	Eosinophils	SSC12: 35,308,994 bp – 37,308,994 bp	*MIR21* (36,065,267 bp – 36,065,358 bp)
SNP4	Monocytes	SSC2: 119,341,201 bp – 121,341,201 bp	*COMMD10* (120,238,623 bp – 120,429,913 bp), *ATG12* (119,948,443 bp – 119,965,702 bp), *CDO1* (119,928,476 bp – 119,940,425 bp), *TMED7* (119,794,608 bp – 119,804,406 bp), *TICAM2* (119,758,636 bp – 119,760,759 bp)
SNP5	Monocytes	SSC9: 104,461,701 bp – 106,461,701 bp	*NAMPT* (106,121,909 bp – 106,161,841 bp)
SNP6	Red blood cells	SSC4: 90,591,493 bp – 92,591,493 bp	*SPTA1* (91,485,067 bp – 91,640,063 bp), *MNDA* (91,416,410 bp – 91,433,243 bp), *ACKR1* (91,221,889 bp – 91,225,651 bp)
SNP7	Red blood cells	SSC6: 27,511,423 bp – 29,511,423 bp	*CBFB* (27,684,030 bp – 27,776,751 bp), *THAP11* (28,465,458 bp – 28,466,387 bp), *PSMB10* (28,544,910 bp – 28,547,609 bp), *LCAT* (28,550,363 bp – 28,553,512 bp), *SLC12A4* (28,554,162 bp – 28,576,458 bp)
SNP8	Red blood cells	SSC8: 40,156,538 bp – 42,156,538 bp	*PDGFRA* (40,967,493 bp – 41,021,442 bp), *KIT* (41,402,334 bp – 41,492,306 bp)
SNP9	Platelets	SSC17: 58,739,745 bp – 60,739,745 bp	*GNAS* (59,031,820 bp – 59,053,022 bp), *TUBB1* (59,161,420 bp – 59,168,385 bp)
SNP10	Red blood cells	SSC1: 17,792,764 bp – 19,792,764 bp	*STXBP5* (18,345,363 bp – 18,513,252 bp), *RAB32* (18,870,875 bp – 19,097,892 bp)
SNP11	Platelets	SSC5: 63,520,638 bp – 65,520,638 bp	*VWF* (64,517,593 bp – 64,655,938 bp), *CD9* (64,420,177 bp – 64,459,776 bp), *GNB3* (63,863,656 bp – 63,870,396 bp), *PHB2* (63,751,566 bp – 63,756,480 bp)
SNP12	Red blood cells	SSC9: 39,919,049 bp – 41,919,049 bp	*ZBTB16* (41,639,701 bp – 41,836,742 bp)
SNP13	Red blood cells	SSC11: 12,749,336 bp – 14,749,336 bp	*TRPC4* (13,300,556 bp – 13,517,568 bp), *FREM2* (13,959,865 bp – 14,154,246 bp)
SNP14	Red blood cells	SSC12: 21,234,265 bp – 23,234,265 bp	*RARA* (22,047,442 bp – 22,085,674 bp), *THRA* (22,270,062 bp – 22,296,618 bp)

^a^The most significant SNP above the genome-wise *FDR* of 0.05 in each genomic region

^b^The category of traits that associated with candidate genes

## Discussion

### Potential roles of candidate genes

Functional enrichment analyses for the candidate gene lists for CBC traits indicated multiple enriched functions that can be considered as functionally and biologically relevant to white blood cell traits in response to a polymicrobial infectious challenge, and red blood cell and platelet traits that were collected before and after exposure to the challenge, such as inflammatory response, cell growth and proliferation, cell-to-cell signaling and interaction, and hematological system development and function.

The candidate genes in Table 4 have been reported to be relevant to particular types of CBC traits by studies in pigs, human, mice, and rat, which may help us to further understand the functions of these candidate genes related to CBC traits in response to the polymicrobial challenge. Of note, candidate genes *ARHGEF2* (*Rho/Rac guanine nucleotide exchange factor*), *TGFB2* (*transforming growth factor beta 2*), and *MIR21* (*microRNA miR-21*) were identified to be functionally and biologically relevant to eosinophils. The product of *ARHGEF2* regulates the activity of GTPases and has been identified to be highly expressed in eosinophils. GTPases are known to be involved in mediator release from granulocytes, which is a crucial event in the activation of eosinophils and neutrophils during inflammation [23, 24]. *TGFB2* has also been found to be expressed mainly in eosinophils, and greater expression of *TGFB2* has been identified to be associated with persistent eosinophilic inflammation (severe asthma) in human [25]. However, in the polymicrobial challenge, an increase in the number of eosinophils may be associated with parasitic infection (e.g. *Ascaris suum*) rather than respiratory disease. Eosinophils play an important role of killing larvae by releasing the toxic content of their granules as part of the immune response [26]. Thus, further investigations are warranted to investigate the functional relationships between the expression of *TGFB2* and response to the challenge. Expression of *MIR21* has not been identified in eosinophils but in other white blood cells, including lymphocytes, monocytes, macrophages, and dendritic cells, which work collaboratively with eosinophils in the immune response [27–29]. Although the mRNA targets for *MIR21* are complex and remain an area of active investigation, it has been demonstrated that *MIR21* acts as a key signal mediating the balance of the inflammatory reaction to promote healing, resolution, and a return to homeostasis [27].

For the candidate genes on SSC2, the product of *COMMD10* has been found to be related to the function of phagosomes in murine macrophages, which promotes phagolysosome maturation and facilitates the timely killing of pathogens [30, 31]. The product of *ATG12* (*autophagy related 12*) is involved in autophagy of circulating monocytes for degradation and recycling of cellular components, which prevents apoptosis (programmed cell death) of monocytes and is essential for monocyte-macrophage differentiation and cytokine production in the innate immune response [32, 33]. The product of *CDO1*, cysteine dioxygenase type 1, catalyzes taurine synthesis and it is commonly accepted that taurine plays an important role in the immune system as an antioxidant to protect phagocytes, including macrophages, from oxidative stress caused by the generation of reactive oxygen species at the site of inflammation [34–37]. Both *TMED7* and *TICAM2* are immunity genes and their products are involved in the function of toll-like receptors (TLRs), which are expressed on macrophages and monocytes and are responsible for the sensing of pathogen-associated-molecular-patterns in the extracellular environment and in endosomes [38–40]. Of note, overexpression of *TMED7* has been found to be associated with inhibition of MyD88-independent TLR4 signaling and the protein encoded by *TICAM2* has been identified as a bridge adaptor recruiting TLRs to mediate innate immune responses [38–40]. In addition, *NAMPT* (*nicotinamide phosphoribosyl transferase*) on SSC9 has been found to be functionally and biologically related to monocytes, and its gene product has been found to play an important role in governing monocyte recruitment and in monocyte-macrophage differentiation [41, 42].

For red blood cells, the majority of candidate genes reported here have been identified as key components involved in hematopoiesis and erythropoiesis responsible for the differentiation and development of red blood cells, including *MNDA* (*myeloid cell nuclear differentiation antigen*) on SSC4, *CBFB* (*core-binding factor subunit beta*) and *THAP11* on SSC6, *PDGFRA* and *KIT* (*KIT proto-oncogene, receptor tyrosine kinase*) on SSC8, and *RARA* (*retinoic receptor alpha*) and *THRA* (*thyroid hormone receptor alpha*) on SSC12 [43–52]. In addition, *SPTA1* on SSC4 encodes a protein in the red blood cell membrane, the products of *LCAT* and *SLC12A4* on SSC6 regulate the lipid composition in the red blood cell membrane and cell swelling, respectively, and all these gene products work together to maintain the normal volume and biconcave shape of red blood cells, which helps to ensure the biological and biomechanical functions of the cells [53–57]. *ACKR1* (*atypical chemokine receptor 1*) on SSC4 and *PSMB10* on SSC6 are candidate genes that have been shown to be involved in the immune response of red blood cells. The receptor ACKR1 expressed in red blood cells was found to regulate immune responses by interacting with chemokines, and which works as a blood-based chemokine buffer involved with the uptake and degradation of chemokines [58]. Meanwhile, ACKR1 has also been identified as an essential regulator of hematopoiesis and erythropoiesis promoting interactions between nuclear progenitor red blood cells and hematopoietic stem cells in the bone marrow [58, 59]. PSMB10 is found to be responsible for intracellular protein degradation and generation of peptides that bind to class I major histocompatibility complex (MHC) molecules [60]. The MHC molecules display these peptides to cytotoxic CD8^+^ T cells to support their activity of immune surveillance [61]. Further, through a study of anemia caused by congenital red blood cell aplasia in human, PSMB10 has been suggested to be functional in the MHC class I machinery in mature red blood cells in response to inflammatory signaling [62].

Candidate genes for platelet traits were annotated into two major functions, platelet aggregation and megakaryopoiesis. Platelet aggregation involves platelet-to-platelet adhesion, which is essential for effective hemostasis following injury and bleeding, and megakaryopoiesis is the process of differentiation and development of platelets [63]. Among them, *CD9* (*CD9 antigen*) on SSC5 encodes a major platelet cell surface glycoprotein and plays dual roles in megakarypoiesis and platelet aggregation [64–67]. The products of *VWF* (*von Willebrand factor*), *PHB2* (*prohibitin 2*), and *GNB3* (*G protein subunit beta 3*) on SSC5 and *GNAS* (*guanine nucleotide binding protein*) on SSC17 were found to be involved in platelet aggregation [68–73]. In addition to megakaryopoiesis, tubulin beta class VI coded by *TUBB1 (tubulin beta 1 class VI)* on SSC17 has been reported to play a role in maintaining platelet morphology [74–76].

### Overlap with previously discovered QTL

In addition to the novel QTL for CBC traits identified in this study, some of the QTL identified have been previously reported. QTL on SSC8 located nearby the *KIT* gene were found to be associated with MCH, MCV, and RBC in this study. In addition, this region has also been identified to show a significant effect on the levels of NEU and HCT in the crossbreds of European Wild Boar × Yorkshire and Landrace × Yorkshire subsequent to stress and disease challenges [15, 22]. For QTL on SSC5 that associated with PLT traits here, Reiner et al. [17] found them to be associated with red blood cell traits in Pietrain × Meishan pigs including HCT, HGB, and RBC traits. These results may be caused by the common myeloid progenitors for all cells mentioned above. Moreover, it may also further indicate the pleiotropic roles of QTL involved in the functions of different blood cells. Apart from studies in pigs, the candidate gene *SPTA1* associated with MCHC has also been identified by GWAS for red blood cell traits in human, which also functions in maintaining the shape and deformability of human red blood cells [77].

### Potential links with disease resilience

Although the QTL uncovered for blood cell traits have small effects in this study, which has also been found in previous GWAS for blood cell traits of pigs and human [21, 77], the genes involved in these QTL are suggested to be involved in hematopoiesis and immune responses in the face of a polymicrobial infectious challenge. In turn, they may contribute to disease resilience, as hematopoiesis and immune response are collaborative mechanisms that play essential roles in defending against pathogens, maintaining homeostasis, and preventing death from the infection [6, 78, 79]. None of the QTL identified for the CBC traits were pleiotropic with GFGR or TR in response to the challenge. However, some candidate genes are known to have pleiotropic effects among different CBC traits and play roles in both hematopoiesis and immune response. For example, *KIT* may be a pleiotropic gene for multiple blood cell populations in response to stress and disease challenge, and *ACKR1* exhibits pleiotropic effects on hematopoiesis and immune responses, as discussed above [15, 22, 58, 59]. Accordingly, these results highlight the importance of further investigating and validating the function of such pleiotropic genes in disease resilience.

## Conclusions

In this study, we identified 14 genomic regions that were significantly associated (genome-wise *FDR < 0.05*) with CBC traits collected from the natural polymicrobial challenge model, including five for white blood cell traits and nine for red blood cell and platelet traits. Candidate genes or regions located nearby significant SNPs were found to have potential roles in immune response pathways, red blood cell morphology, platelet aggregation, and hematopoiesis, including granulopoiesis and granulocytic differentiation, erythropoiesis, and megakarypoiesis. These results complement previous GWAS for blood cell traits in pigs and contribute to improving our understanding of the genetic basis of blood cell composition before and after exposure to a polymicrobial infectious challenge. This study also advances understanding of the genetic control of disease resilience, as blood cells are key players in an animal’s immune response and are recruited by hematopoiesis. Validation and identification of the candidate genes and causal mutations are necessary to further investigate and develop the use of CBC traits to enhance genetic improvement of disease resilience for the pig industry.

## Methods

### Natural disease challenge model and phenotypic traits

Details of the natural disease challenge model (NDCM) and the collection of phenotypic traits are described in Bai et al. [10] and Putz et al. [80]. Briefly, the NDCM was established to simulate a polymicrobial infectious challenge and severe disease pressure often found at the commercial level of pig production. A 3-week healthy quarantine nursery after weaning and a test station that consisted of a 4-week second-stage nursery and an approximately 16-week grow-to-finish stage were the two main facilities in the NDCM. A total of 2743 healthy F1 crossbred (Landrace × Yorkshire) barrows owned by company Centre de Développement du Porc du Québec, Inc. were introduced into the NDCM in 42 batches at 3-week intervals after weaning at an average age of 21 days old. Each batch consisted of 60 or 75 weaned crossbred barrows that were provided by one of the seven members of PigGen Canada from healthy multiplier farms. All pigs from each batch were sourced from one multiplier farm, and over time, 13 farms were involved, and each supplied one to six batches of pigs. Pigs that went through the NDCM were first exposed to the polymicrobial infectious challenge in the second-stage nursery. The challenge was established by co-introducing four groups of 12 to 28 commercial seeder pigs from three strategically selected commercial farms with known disease outbreaks together with the first four batches of healthy barrows into the NDCM. Common disease-causing pathogens found in commercial farms were the major target pathogens in the NDCM, including multiple strains of porcine reproductive and respiratory syndrome virus (PRRSV) and swine influenza A virus, various respiratory and enteric bacterial pathogens (such as *Mycoplasma hyopneumoniae*, *Haemophilus parasuis*, *Brachyspira hampsonii*, *Salmonella enterica* serovar typhimurium, and *Streptococcus suis*), and two parasites (*Cystoisospora suis* and *Ascaris suum*). Subsequently, the challenge model was maintained as a continuous flow system by nose-to-nose direct contact between the new batch of healthy barrows and the preceding challenged group during the first week of challenge nursery period, except during the period of excessively high challenge pressure when indirect contact was used to help maintain the mortality rate below the target level established by the Animal Protection Committee. Serum samples were randomly collected from a subset of individuals for RT-PCR 4 weeks post-challenge and ELISA 6 weeks post-challenge to confirm that every batch had been exposed to PRRSV in the test station and to monitor the pathogens in the test station for each batch. The disease pressure varied by batch; not all pigs were exposed to all the same pathogens because not all pathogens were identified in all batches, as would be the case on a commercial farm. Clinical signs and mortality were monitored in the NDCM for treatment decisions made by herd veterinarians and trained staff, including individual treatments that were given on a case-by-case basis and group medication through water and feed on a batch-level, as necessary, to maintain a balance between disease pressure and animal welfare. Of a total of 2743 pigs, some of the animals died due to infectious diseases after exposure to the challenge (*n* = 561), a few animals died due to non-infectious and unclear reasons (*n* = 164) and their phenotypes were set to missing, the other animals (*n* = 2018) reaching the target slaughter weight at approximately 181 days old were slaughtered commercially and entered the food chain after the study.

In total, four sets of blood samples (Blood 1, Blood 2, Blood 3, and Blood 4) were collected from the jugular vein. Blood 2 was collected immediately before entry into the challenge nursery and polymicrobial infectious challenge at 40 days of age. Whole EDTA-anticoagulated blood was collected for CBC analyses at Blood 1, Blood 3, and Blood 4 (2-weeks before, and at 2- and 6-weeks after exposure to the challenge) at an average age of 26 days, 54 days, and 82 days, respectively, using the ADVIA® 2120i Hematology System (Siemens Healthineer, Erlangen, Germany) within 24 to 48 h of collection. The standard deviation of the bleeding age was 2, 3, and 3 days for Blood 1, Blood 3, and Blood 4, respectively. CBC traits used for this study were described and the outliers were removed previously [10]. There are three categories of CBC traits: (1) six white blood cell traits, including total white blood cell concentration (WBC, 10^3^/μL), neutrophil concentration (NEU, 10^3^/μL), lymphocyte concentration (LYM, 10^3^/μL), monocyte concentration (MONO, 10^3^/μL), eosinophil concentration (EOS, 10^3^/μL), and basophil concentration (BASO, 10^3^/μL); (2) seven red blood cell traits, consisting of red blood cell concentration (RBC, 10^6^/μL), hemoglobin concentration (HGB, g/L), hematocrit (HCT, %), mean corpuscular volume (MCV, fL), mean corpuscular hemoglobin (MCH, pg), mean corpuscular hemoglobin concentration (MCHC, g/L), and red blood cell distribution width (RDW, %); and (3) two platelet traits, including platelet concentration (PLT, 10^3^/μL) and mean platelet volume (MPV, fL). CBC traits were assessed at individual time points (Blood 1, Blood 3, Blood 4) as well as calculating and testing the changes between time points: Blood 1 to Blood 3 (Δ 13, calculated as Blood 3 − Blood 1), Blood 3 to Blood 4 (Δ 34, Blood 4 − Blood 3), and Blood 1 to Blood 4 (Δ 14, Blood 4 − Blood 1). All white blood cell traits in Blood 1, Blood 3, and Blood 4 were log_10_-transformed to reduce skewness of the distribution.

Body weights and veterinary treatments were recorded on an individual pig basis and used to calculate production traits, grow-to-finish growth rate (GFGR), and treatment rate (TR), which were regarded as economically important traits related to disease resilience. The GFGR for each animal was estimated using linear regression of body weights measured every 3 weeks in the grow-to-finish phase on ages from an average age of 69 days of age to the endpoint, i.e., either mortality due to infectious diseases or reaching the target slaughter weight at approximately 181 days old. The TR for each animal was the number of individual treatment events given on a case-by-case basis standardized by the number of days the animal spent in the NDCM (TR = number of treatment events/days × 100%). The TR for animals that died before receiving any treatment was set to missing.

### SNP array genotyping and quality control

The genotyping using the 650 K Affymetrix Axiom® Porcine Genotyping Array was performed at Delta Genomics (Edmonton AB, Canada). Raw Affymetrix SNP data were processed by Delta Genomics with the Axiom Analysis Suite, using all defaults (sample call rate ≥ 97%; SNP call rate ≥ 97%; number of minor alleles observed ≥2). Imputation of sporadic missing genotypes was completed using FImpute [81]. The pedigree was utilized for imputation but only included the dam, since sire was typically unknown due to the use of pooled semen. The preGSf90 software in the BLUPF90 suite of programs was used to remove SNPs with minor allele frequency lower than 0.01 [82]. After quality control, there were 2593 genotyped animals with 465,910 autosomal SNPs remained for the subsequent analyses.

### Population stratification and linkage disequilibrium estimation

Population stratification among genotyped animals was investigated using PLINK 1.90 [83] based on pairwise identity-by-state (IBS) distance, which was estimated using SNP genotypes. A multidimensional scaling (MDS) plot based on IBS pairwise distance was drawn by the ‘ggplot2’ package in R [84] to show the first three dimensions of the population structure. The genomic inflation factor and quantile–quantile (Q–Q) plots were applied to assess genomic inflation of the test statistics using the R packages of ‘GenABEL’ and ‘qqman’ [84–86]. The linkage disequilibrium (LD) of pairwise SNPs was measured as the squared correlation (r^2^) of allele counts for the two SNPs and haplotype blocks were built using the Haploview software [87, 88].

### Single-step GWAS and models

Univariate and multivariate single-step GWAS (SSGWAS) for CBC traits were implemented in the BLUPF90 suite of programs [82, 89] with the joint pedigree-genomic relationship matrix (**H**) for single-marker associations, accommodating both genotyped (*n* = 2593) and non-genotyped (*n* = 150) animals. Details for algorithms employed for these analyses have been described by Aguilar et al. [89]. Briefly, BLUPF90 combines the algorithms for single-step GBLUP and for back-solving to obtain estimates and *p*-values for SNP associations from estimates of breeding values. The genomic relationship matrix (***G***) for genotyped animals was constructed as **ZZ**^′^/2 ∑ p_i_(1 − p_i_), where the **Z** matrix contains centered SNP genotype codes and p_i_ is the minor allele frequency for SNP *i* [90]. The p-values for SNP associations were adjusted for multiple testing by the Benjamini and Hochberg correction (false discovery rate, *FDR*) [84, 91]. An *FDR* threshold of 0.05 was used to control false positive results and to declare significant associations. The most significant SNP above the genome-wise *FDR* of 0.05 in each genomic region were referred to as the top significant SNP, which were further separated into top lead and top floating SNPs, which referred to top significant SNPs in a genomic region with or without a group of supportive SNPs, respectively.

The univariate mixed linear model used for GWAS can be described as follows:

$$ \mathbf{y}=\mathbf{Xb}+\mathbf{Za}+\mathbf{Wc}+\mathbf{e} $$

where ***y*** is a vector of observations on a CBC trait for all individuals, **b** is a vector of fixed effects, including the effect of batch and the covariate of bleeding age, **X** is a design matrix relating observations to the fixed effects, **a** is a vector of breeding values, **Z** is a design matrix that relates observations to breeding values, including genotyped and ungenotyped animals, and ***e*** is a vector of residual effects. Vector **c** represents a stack of vectors (**c**_**Litter**_, **c**_**Pen1**_, **c**_**Pen2**_, and **c**_**Pen3**_) of independent and uncorrelated random environmental effects, including litter (**c**_**Litter**_) and pen effects in the quarantine unit (**c**_**Pen1**_), in the test station second-stage nursery (**c**_**Pen2**_**)**, and in the test station grow-to-finish stage (**c**_**Pen3**_). These random environmental effects were tested and fitted in the model for each CBC trait when they were significant (*p < 0.05*). Matrix **W** (**W**_**Litter**_, **W**_**Pen1**_, **W**_**Pen2**_, and **W**_**Pen3**_) is a stack of incidence matrices that relate observations to the corresponding random environmental effects. The random effects fitted for each of CBC traits were the same as Bai et al. [10].

Assuming the random effects **c** and **e** are uncorrelated and identically distributed, the (co-)variances of random effects for univariate models are:

$$ \operatorname{var}\left[\begin{array}{c}\mathbf{a}\\ {}\mathbf{c}\\ {}\mathbf{e}\end{array}\right]=\left[\begin{array}{ccc}{\mathbf{H}\upsigma}_{\mathrm{a}}^2& 0& 0\\ {}0& {\mathbf{I}\boldsymbol{\upsigma}}_{\mathbf{c}}^{\mathbf{2}}& 0\\ {}0& 0& {\mathbf{I}\upsigma}_{\mathrm{e}}^2\end{array}\right] $$

where **H** is the joint pedigree-genomic relationship matrix for genotyped and non-genotyped animals as mentioned above, **I** is the identity matrix, ${\upsigma}_{\mathrm{a}}^2$ is the additive genetic variance, ${\boldsymbol{\upsigma}}_{\mathbf{c}}^{\mathbf{2}}$ represents a stack of random effect variances (e.g. $$ {\boldsymbol{\upsigma}}_{\mathbf{c}}^{\mathbf{2}}=\left[\begin{array}{cc}{\upsigma}_{{\mathrm{c}}_{\mathrm{Litter}}}^2& 0\\ {}0& {\upsigma}_{{\mathrm{c}}_{\mathrm{Pen}1}}^2\end{array}\right] $$**,** when the random effects **c**_**Litter**_ and **c**_**Pen1**_ are significant and fitted in the model for a trait), and ${\upsigma}_{\mathrm{e}}^2$ is the residual variance.

The model for multivariate analyses resembles a stack of univariate models for each of the traits that were found to be highly genetic correlated in [10], which can be written as [11, 92]:

$$ \left[\begin{array}{c}{\mathbf{y}}_{\mathbf{1}}\\ {}{\mathbf{y}}_{\mathbf{2}}\\ {}{\mathbf{y}}_{\mathbf{3}}\end{array}\right]=\left[\begin{array}{c}{\mathbf{X}}_{\mathbf{1}}{\mathbf{b}}_{\mathbf{1}}+{\mathbf{Z}}_{\mathbf{1}}{\mathbf{a}}_{\mathbf{1}}+{\mathbf{W}}_{\mathbf{1}}{\mathbf{c}}_{\mathbf{1}}+{\mathbf{e}}_{\mathbf{1}}\\ {}{\mathbf{X}}_{\mathbf{2}}{\mathbf{b}}_{\mathbf{2}}+{\mathbf{Z}}_{\mathbf{2}}{\mathbf{a}}_{\mathbf{2}}+{\mathbf{W}}_{\mathbf{2}}{\mathbf{c}}_{\mathbf{2}}+{\mathbf{e}}_{\mathbf{2}}\\ {}{\mathbf{X}}_{\mathbf{3}}{\mathbf{b}}_{\mathbf{3}}+{\mathbf{Z}}_{\mathbf{3}}{\mathbf{a}}_{\mathbf{3}}+{\mathbf{W}}_{\mathbf{3}}{\mathbf{c}}_{\mathbf{3}}+{\mathbf{e}}_{\mathbf{3}}\end{array}\right] $$

For each trait in the multivariate model, the same effects were fitted as in the univariate models. For multivariate models, assuming random effects **c**_**n**_ and residual effects **e**_**n**_ for the n^th^ trait (*n* = 1, 2, 3) are uncorrelated and identically distributed, the (co-) variances of random effects are:

$$ \mathrm{Var}\left[\begin{array}{c}{\mathbf{a}}_{\mathbf{1}}\\ {}{\mathbf{a}}_{\mathbf{2}}\\ {}{\mathbf{a}}_{\mathbf{3}}\\ {}{\mathbf{c}}_{\mathbf{1}}\\ {}{\mathbf{c}}_{\mathbf{2}}\\ {}{\mathbf{c}}_{\mathbf{3}}\\ {}{\mathbf{e}}_{\mathbf{1}}\\ {}{\mathbf{e}}_{\mathbf{2}}\\ {}{\mathbf{e}}_{\mathbf{3}}\end{array}\right]=\left[\begin{array}{ccccccccc}{\mathbf{H}\upsigma}_{{\mathrm{a}}_1}^2& {\mathbf{H}\upsigma}_{{\mathrm{a}}_{12}}& {\mathbf{H}\upsigma}_{{\mathrm{a}}_{13}}& 0& 0& 0& 0& 0& 0\\ {}{\mathbf{H}\upsigma}_{{\mathrm{a}}_{21}}& {\mathbf{H}\upsigma}_{{\mathrm{a}}_2}^2& {\mathbf{H}\upsigma}_{{\mathrm{a}}_{23}}& 0& 0& 0& 0& 0& 0\\ {}{\mathbf{H}\upsigma}_{{\mathrm{a}}_{31}}& {\mathbf{H}\upsigma}_{{\mathrm{a}}_{32}}& {\mathbf{H}\upsigma}_{{\mathrm{a}}_3}^2& 0& 0& 0& 0& 0& 0\\ {}0& 0& 0& {\mathbf{I}\boldsymbol{\upsigma}}_{{\mathbf{c}}_{\mathbf{1}}}^{\mathbf{2}}& {\mathbf{I}\boldsymbol{\upsigma}}_{{\mathbf{c}}_{\mathbf{1}\mathbf{2}}}& {\mathbf{I}\boldsymbol{\upsigma}}_{{\mathbf{c}}_{\mathbf{1}\mathbf{3}}}& 0& 0& 0\\ {}0& 0& 0& {\mathbf{I}\boldsymbol{\upsigma}}_{{\mathbf{c}}_{\mathbf{2}\mathbf{1}}}& {\mathbf{I}\boldsymbol{\upsigma}}_{{\mathbf{c}}_{\mathbf{2}}}^{\mathbf{2}}& {\mathbf{I}\boldsymbol{\upsigma}}_{{\mathbf{c}}_{\mathbf{2}\mathbf{3}}}& 0& 0& 0\\ {}0& 0& 0& {\mathbf{I}\boldsymbol{\upsigma}}_{{\mathbf{c}}_{\mathbf{3}\mathbf{1}}}& {\mathbf{I}\boldsymbol{\upsigma}}_{{\mathbf{c}}_{\mathbf{3}\mathbf{2}}}& {\mathbf{I}\boldsymbol{\upsigma}}_{{\mathbf{c}}_{\mathbf{3}}}^{\mathbf{2}}& 0& 0& 0\\ {}0& 0& 0& 0& 0& 0& {\mathbf{I}\upsigma}_{{\mathrm{e}}_1}^2& {\mathbf{I}\upsigma}_{{\mathrm{e}}_{12}}& {\mathbf{I}\upsigma}_{{\mathrm{e}}_{13}}\\ {}0& 0& 0& 0& 0& 0& {\mathbf{I}\upsigma}_{{\mathrm{e}}_{21}}& {\mathbf{I}\upsigma}_{{\mathrm{e}}_2}^2& {\mathbf{I}\upsigma}_{{\mathrm{e}}_{23}}\\ {}0& 0& 0& 0& 0& 0& {\mathbf{I}\upsigma}_{{\mathrm{e}}_{31}}& {\mathbf{I}\upsigma}_{{\mathrm{e}}_{32}}& {\mathbf{I}\upsigma}_{{\mathrm{e}}_3}^2\end{array}\right] $$

where ${\upsigma}_{{\mathrm{a}}_{12}}={\upsigma}_{{\mathrm{a}}_{21}}$, ${\upsigma}_{{\mathrm{a}}_{13}}={\upsigma}_{{\mathrm{a}}_{31}}$, and ${\upsigma}_{{\mathrm{a}}_{23}}={\upsigma}_{{\mathrm{a}}_{32}}$ are additive genetic covariances between traits, ${\boldsymbol{\upsigma}}_{{\mathbf{c}}_{\mathbf{12}}}={\boldsymbol{\upsigma}}_{{\mathbf{c}}_{\mathbf{21}}}$, ${\boldsymbol{\upsigma}}_{{\mathbf{c}}_{\mathbf{13}}}={\boldsymbol{\upsigma}}_{{\mathbf{c}}_{\mathbf{31}}}$, and ${\boldsymbol{\upsigma}}_{{\mathbf{c}}_{\mathbf{23}}}={\boldsymbol{\upsigma}}_{{\mathbf{c}}_{\mathbf{32}}}$ are covariances for common random effects between two traits, ${\upsigma}_{{\mathrm{e}}_{12}}={\upsigma}_{{\mathrm{e}}_{21}}$, ${\upsigma}_{{\mathrm{e}}_{13}}={\upsigma}_{{\mathrm{e}}_{31}}$, and ${\upsigma}_{{\mathrm{e}}_{23}}={\upsigma}_{{\mathrm{e}}_{32}}$ are covariances for residual effects between two traits.

### Post-GWAS marker effect analyses

The percentage of additive genetic variance explained by a 1 Mb window (with a median of 224 adjacent SNPs) was estimated by conducting window-based inferences for additive genetic variance in the BLUPF90 suit of programs [82]. Each chromosome was evaluated by using a sliding (moving) 1 Mb window by using every SNP on the chromosome as a starting SNP for a window segment [82]. Therefore, a top significant SNP was contained within multiple windows and among them, the largest percentage of additive genetic variance explained by a window that contained that top significant SNP was reported for each trait.

Additive and dominance effects of each top significant SNP were estimated using the BLUPF90 suite of programs [82] based on the following model:

$$ \mathbf{y}=\mathbf{Xb}+\mathbf{Za}+\mathbf{Wc}+\mathbf{v}\upalpha +\mathbf{d}\updelta +\mathbf{e} $$

where **y**, **X**, **b**, **Z**, **a**, **W**, **c**, and **e** are the same as for the univariate model described above; **v** is a vector of the top significant SNP genotypes coded as − 1, 0, and 1 for the AA, AB, and BB, respectively; α is the additive effect; **d** is a vector of dominance coded as 1 for heterozygous genotype (AB) and 0 for homozygous genotypes (AA and BB); δ is the dominance effect. Vectors **v** and **d** were fitted as covariates and the top significant SNPs were fitted one by one in the model. The likelihood ratio test was used to test the significance of the additive and dominance effects for each of the top significant SNPs by comparing full models to restricted models that constrained additive or dominance effects to zero using the REMLF90 program of BLUPF90 [82]. When the dominance effect was not significant (*p > 0.05*), the additive effect for a SNP was re-estimated by removing the dominance effect from the model.

### Post-GWAS bioinformatics analyses

Ingenuity Pathway Analysis (IPA) (Ingenuity® Systems, Redwood City, CA; https://www.qiagenbioinformatics.com/products/ingenuity-pathway-analysis/, IPA Spring 2020 release) was used for functional enrichment analyses of candidate genes in significant genomic regions for the CBC trait. A maximum distance of 1 Mb on either side of the lead SNPs based on a genome-wise *FDR < 0.10* was used to search for candidate genes for white blood cell traits of EOSB3 and MONOΔ13. The lead pleiotropic SNPs at genome-wise *FDR < 0.10* were used to search for common candidate genes located within 1 Mb on either side of the SNPs for red blood cell (MCH, MCV, and RBC) and (MPV and PLT) platelet traits in different time points before and after exposure to the challenge. A relaxed *FDR < 0.10* threshold for associated SNPs was used here to increase identification of true positives for the significance of biological and functional relevance of candidate genes [93]. Identification of positional candidate genes was conducted using the UCSC Genome Browser for the Ensembl annotation of the Sscrofa11.1 build of the swine genome (https://genome.ucsc.edu). One collective gene list was created for each trait by combing all candidate genes in associated genomic regions for IPA [94, 95]. Human, mouse, and rat genes in the IPA knowledge base database were used as background for biological function analyses in diseases, molecular and cellular functions, and physiological system development and function categories. A biological function was considered significantly enriched if the *p*-value for the overlap comparison test between the input list of candidate genes and the IPA database was less than 0.05 [94–96].

## Supplementary Information

**Additional file 1: Figure S1.** Violin plots for descriptive statistics for white blood cell traits in Blood 1, Blood 3, and Blood 4 collected at 2-weeks before, and at 2- and 6-weeks after the challenge, respectively. **Figure S2.** Violin plots for descriptive statistics for red blood cell traits in Blood 1, Blood 3, and Blood 4 collected at 2-weeks before, and at 2- and 6-weeks after the challenge, respectively. **Figure S3.** Violin plots for descriptive statistics for platelet traits in Blood 1, Blood 3, and Blood 4 collected at 2-weeks before, and at 2- and 6-weeks after the challenge, respectively. **Table S1.** Genetic correlations between red blood cell traits of MCH, MCV, and RBC traits in Blood 1, Blood 3, and Blood 4.

**Additional file 2: Figure S4.** Multidimensional scaling (MDS) plots showing the first three dimensions (C1, C2, and C3) of the population structure for genotyped animals based on pairwise identity-by-state distance. **Figure S5.** (A) Manhattan plot for EOSB3. (B) Quantile-Quantile plot for EOSB3; **Figure S6**. (A) Manhattan plot for MONO ∆ 13. (B) Quantile-Quantile plot for MONO ∆ 13; **Figure S7.** Manhattan plots (A, C, E) and Quantile-Quantile plots (B, D, F) for MCH in Blood 1, Blood 3, and Blood 4, respectively; **Figure S8.** Manhattan plots (A, C) and Quantile-Quantile plots (B, D) for MCHC in Blood 3 and for the change of MCHC from Blood 1 to Blood 4, respectively; **Figure S9.** Manhattan plots (A, C, E) and Quantile-Quantile plots (B, D, F) for MCV in Blood 1, Blood 3, and Blood 4, respectively; **Figure S10.** Manhattan plots (A, C, E) and Quantile-Quantile plots (B, D, F) for RBC in Blood 1, Blood 3, and Blood 4, respectively; **Figure S11.** Manhattan plots (A, C) and Quantile-Quantile plots (B, D) for MPV in Blood 1 and Blood 4, respectively; **Figure S12.** Manhattan plots (A, C, E) and Quantile-Quantile plots (B, D, F) for PLT in Blood 1, Blood 3, and Blood 4, respectively.

**Additional file 3: Table S2.** Browsing regions for candidate genes located within 1-Mb on either side of lead SNPs (*FDR* < 0.10) associated with complete blood cell count traits; **Figure S13.** Haplotype block pattern (r^2^-scheme) for the region of candidate genes on SSC2 located within the maximum distance of 1 Mb on either side of the top lead SNP4 (SSC2, 120,341,201 bp); **Figure S14.** Haplotype block pattern (r^2^-scheme) for the region of candidate genes on SSC4 located within the maximum distance of 1 Mb on either side of the top lead SNP6 (SSC4, 91,591,493 bp); **Figure S15.** Haplotype block pattern (r^2^-scheme) for the region of candidate genes on SSC6 located within the maximum distance of 1 Mb on either side of the top lead SNP7 (SSC6, 28,511,423 bp); **Figure S16.** Haplotype block pattern (r^2^-scheme) for SNPs (40,946,144 bp to 41,198,574 bp) on SSC8 located within the maximum distance of 1 Mb on either side of the top lead SNP8 (SSC8, 41,156,538 bp).

**Additional file 4. **This file contains a list of top five enriched biological functions (*p*-value < 0.05) for the candidate gene lists for EOSB3, MONO ∆ 13, and for MCH, MCV, RBC, MPV, and PLT in Blood 1, Blood 3, and Blood 4.

## Data Availability

The data that support the findings of this study were generated on samples from commercially owned animals provided by members of the PigGen Canada consortium. Restrictions apply to the availability of these data, which were used under license for the current study, and so are not publicly available. Data are however available from the corresponding author GP upon non-commercial use and reasonable request and with permission of the PigGen Canada consortium.

## Abbreviations

BASO: Basophil concentration
CBC: Complete blood count
DNA: Deoxyribonucleic acid
EOS: Eosinophil concentration
FDR: Benjamini and Hochberg correction for false discovery rate
GFGR: Grow-to-finish growth rate
GWAS: Genome-wide association studies
HCT: Hematocrit
HGB: Hemoglobin concentration
IBS: Identity-by-state
IPA: Ingenuity Pathway Analysis
LD: Linkage disequilibrium
LYM: Lymphocyte concentration
LSMs: Least-squares means
MCH: Mean corpuscular hemoglobin
MCHC: Mean corpuscular hemoglobin concentration
MCV: Mean corpuscular volume
MDS: Multidimensional scaling
MHC: Major histocompatibility complex
MONO: Monocyte concentration
MPV: Mean platelet volume
NDCM: Natural disease challenge model
NEU: Neutrophil concentration
PCA: Principle component analysis
PLT: Platelet concentration
PRRSV: Porcine reproductive and respiratory syndrome virus
Q-Q plot: Quantile-quantile plot
QTL: Quantitative trait loci
RBC: Red blood cell concentration
RDW: Red blood cell distribution width
SSC: *Sus scrofa* chromosome
SSGWAS: Single-step genome-wide association studies
SNP: Single-nucleotide polymorphisms
TLRs: Toll-like receptors
TR: Treatment rate
WBC: Total white blood cell concentration
