# Supplementary material for: Investigating the genetic architecture of disease resilience in pigs by genome-wide association studies of complete blood count traits collected from a natural disease challenge model
Source: BMC Genomics. 2021 Jul 13;22:535. doi: 10.1186/s12864-021-07835-4 (PMC8278769; doi:10.1186/s12864-021-07835-4)
Supplement: Supplementary file 1 — Additional file 1: Figure S1. Violin plots for descriptive statistics for white blood cell traits in Blood 1, Blood 3, and Blood 4 collected at 2-weeks before, and at 2- and 6-weeks after the challenge, respectively. Figure S2. Violin plots for descriptive statistics for red blood cell traits in Blood 1, Blood 3, and Blood 4 collected at 2-weeks before, and at 2- and 6-weeks after the challenge, respectively. Figure S3. Violin plots for descriptive statistics for platelet traits in Blood 1, Blood 3, and Blood 4 collected at 2-weeks before, and at 2- and 6-weeks after the challenge, respectively. Table S1. Genetic correlations between red blood cell traits of MCH, MCV, and RBC traits in Blood 1, Blood 3, and Blood 4. [file 12864_2021_7835_MOESM1_ESM.pdf]

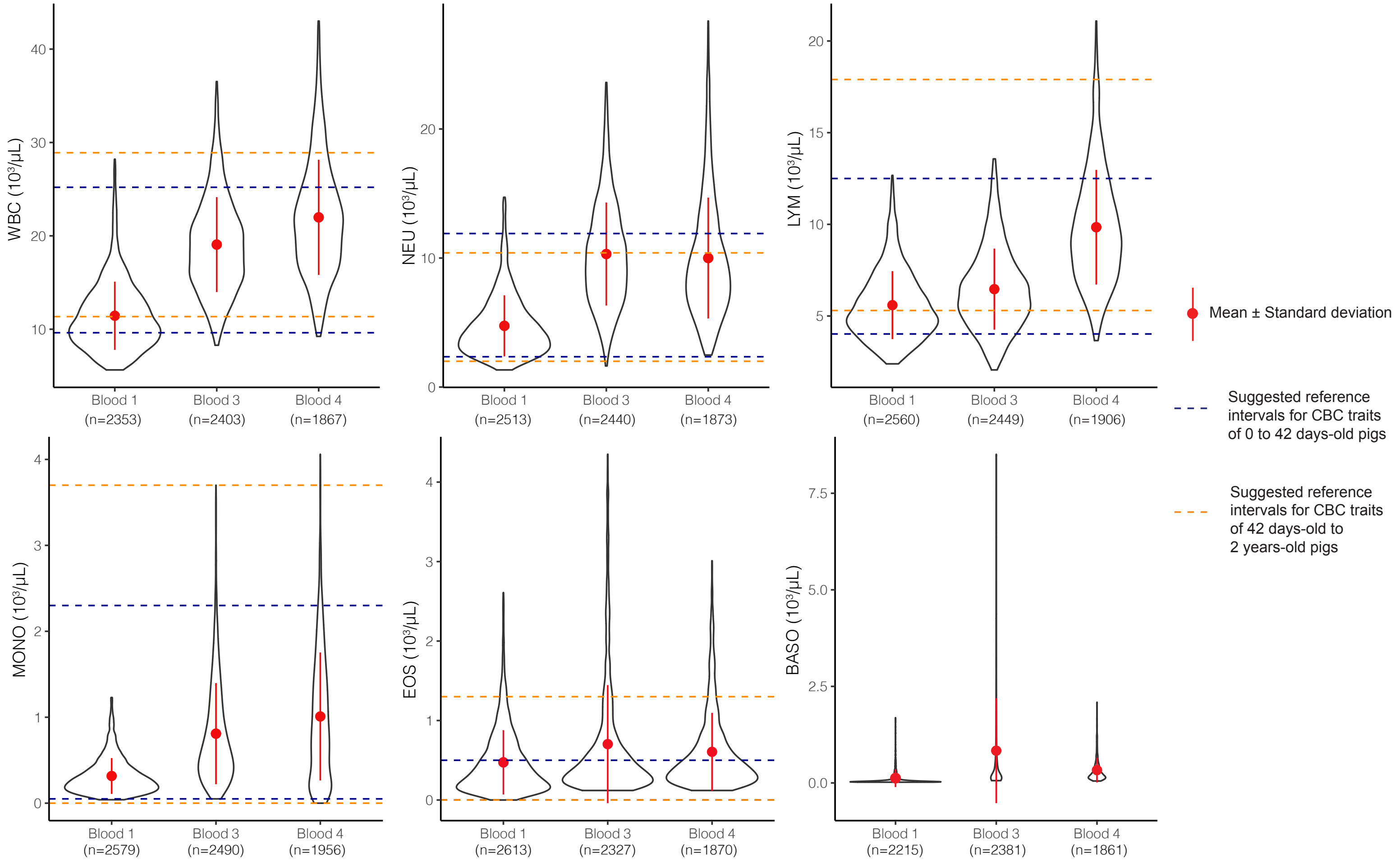


**Figure S1.** Violin plots for descriptive statistics (mean, standard deviation, maximum, minimum and distribution after removing outliers) for white blood cell traits in Blood 1, Blood 3, and Blood 4 collected at 2-weeks before, and at 2- and 6-weeks after the challenge, respectively. WBC: total white blood cell concentration; NEU: neutrophil concentration; LYM: lymphocyte concentration; MONO: monocyte concentration; EOS: eosinophil concentration; BASO: basophil concentration. Wider sections of the violin plots represent a higher probability density of the data at the given value and the skinnier sections represent a lower probability. Suggested reference intervals for CBC traits were derived from Iowa State University's Clinical Pathology Laboratory (2011). The suggested reference intervals for BASO traits are not applicable.

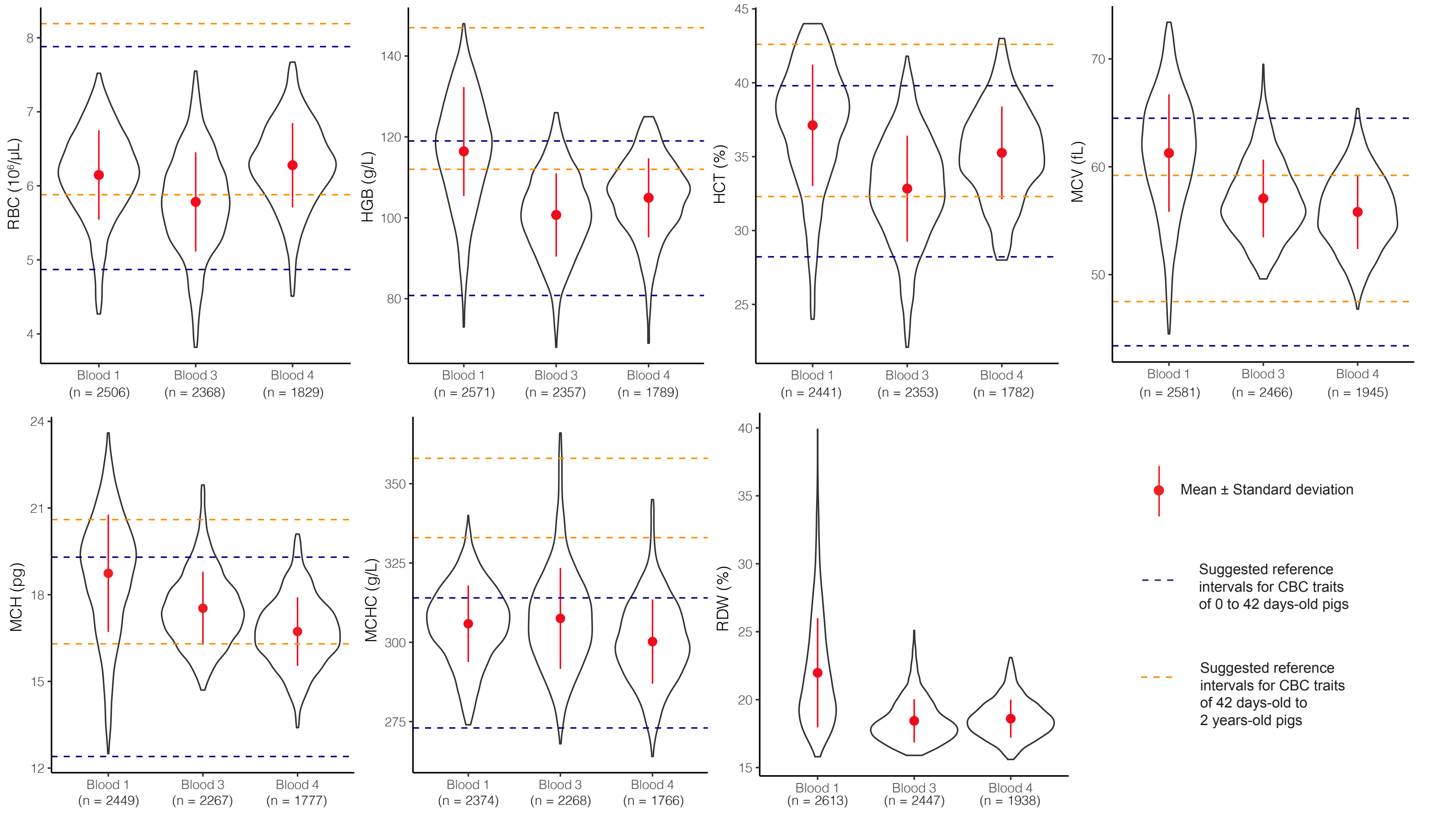


**Figure S2.** Violin plots for descriptive statistics (mean, standard deviation, maximum, minimum and distribution after removing outliers) for red blood cell traits in Blood 1, Blood 3, and Blood 4 collected at 2-weeks before, and at 2- and 6-weeks after the challenge, respectively. RBC: red blood cell concentration; HGB: hemoglobin concentration; HCT: hematocrit; MCV: mean corpuscular volume; MCH: mean corpuscular hemoglobin; MCHC: mean corpuscular hemoglobin concentration; RDW: red blood cell distribution width. Wider sections of the violin plots represent a higher probability density of the data at the given value and the skinnier sections represent a lower probability. Suggested reference intervals for CBC traits were derived from Iowa State University's Clinical Pathology Laboratory (2011). The suggested reference intervals for RDW traits are not applicable.

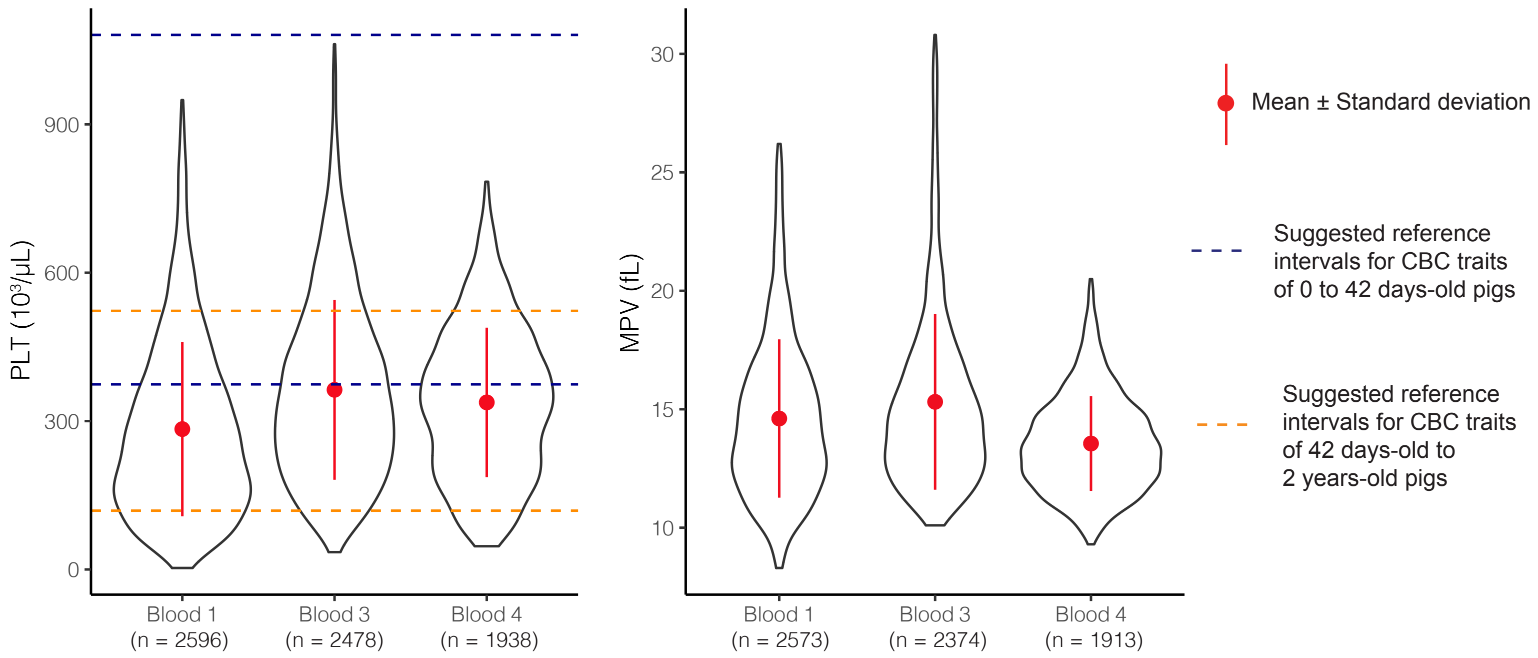


**Figure S3.** Violin plots for descriptive statistics (mean, standard deviation, maximum, minimum and distribution after removing outliers) for platelet traits in Blood 1, Blood 3, and Blood 4 collected at 2-weeks before, and at 2- and 6-weeks after the challenge, respectively. PLT: platelet concentration; MPV: mean platelet volume. Wider sections of the violin plots represent a higher probability that members of the population shown as the given value and the skinnier sections represent a lower probability. Suggested reference intervals for CBC traits were derived from Iowa State University's Clinical Pathology Laboratory (2011). The suggested reference intervals for MPV traits are not applicable.

**Table S1.** Genetic correlations between red blood cell traits of mean corpuscular hemoglobin (MCH), mean corpuscular volume (MCV), and red blood cell concentration (RBC) traits in Blood 1, Blood 3, and Blood 4.

| **Traits** | | **MCH** | | |
|---|---|---|---|---|
| | | **Blood 1** | **Blood 3** | **Blood 4** |
| **RBC** | **Blood 1** | -0.71 ± 0.10 | -0.83 ± 0.07 | -0.60 ± 0.09 |
| | **Blood 3** | -0.62 ± 0.10 | -0.74 ± 0.06 | -0.43 ± 0.09 |
| | **Blood 4** | -0.69 ± 0.10 | -0.66 ± 0.08 | -0.55 ± 0.08 |
| **MCV** | **Blood 1** | 0.90 ± 0.03 | 0.81 ± 0.07 | 0.77 ± 0.08 |
| | **Blood 3** | 0.75 ± 0.07 | 0.86 ± 0.03 | 0.77 ± 0.05 |
| | **Blood 4** | 0.95 ± 0.02 | 0.86 ± 0.04 | 0.95 ± 0.02 |
| **Traits** | | **MCV** | | |
| | | **Blood 1** | **Blood 3** | **Blood 4** |
| **RBC** | **Blood 1** | -0.72 ± 0.09 | -0.68 ± 0.08 | -0.54 ± 0.09 |
| | **Blood 3** | -0.65 ± 0.09 | -0.59 ± 0.08 | -0.44 ± 0.10 |
| | **Blood 4** | -0.71 ± 0.10 | -0.56 ± 0.08 | -0.55 ± 0.08 |
