# Supplementary material for: Investigating the genetic architecture of disease resilience in pigs by genome-wide association studies of complete blood count traits collected from a natural disease challenge model
Source: BMC Genomics. 2021 Jul 13;22:535. doi: 10.1186/s12864-021-07835-4 (PMC8278769; doi:10.1186/s12864-021-07835-4)
Supplement: Supplementary file 2 — Additional file 2: Figure S4. Multidimensional scaling (MDS) plots showing the first three dimensions (C1, C2, and C3) of the population structure for genotyped animals based on pairwise identity-by-state distance. Figure S5. (A) Manhattan plot for EOSB3. (B) Quantile-Quantile plot for EOSB3; Figure S6. (A) Manhattan plot for MONO ∆ 13. (B) Quantile-Quantile plot for MONO ∆ 13; Figure S7. Manhattan plots (A, C, E) and Quantile-Quantile plots (B, D, F) for MCH in Blood 1, Blood 3, and Blood 4, respectively; Figure S8. Manhattan plots (A, C) and Quantile-Quantile plots (B, D) for MCHC in Blood 3 and for the change of MCHC from Blood 1 to Blood 4, respectively; Figure S9. Manhattan plots (A, C, E) and Quantile-Quantile plots (B, D, F) for MCV in Blood 1, Blood 3, and Blood 4, respectively; Figure S10. Manhattan plots (A, C, E) and Quantile-Quantile plots (B, D, F) for RBC in Blood 1, Blood 3, and Blood 4, respectively; Figure S11. Manhattan plots (A, C) and Quantile-Quantile plots (B, D) for MPV in Blood 1 and Blood 4, respectively; Figure S12. Manhattan plots (A, C, E) and Quantile-Quantile plots (B, D, F) for PLT in Blood 1, Blood 3, and Blood 4, respectively. [file 12864_2021_7835_MOESM2_ESM.pdf]

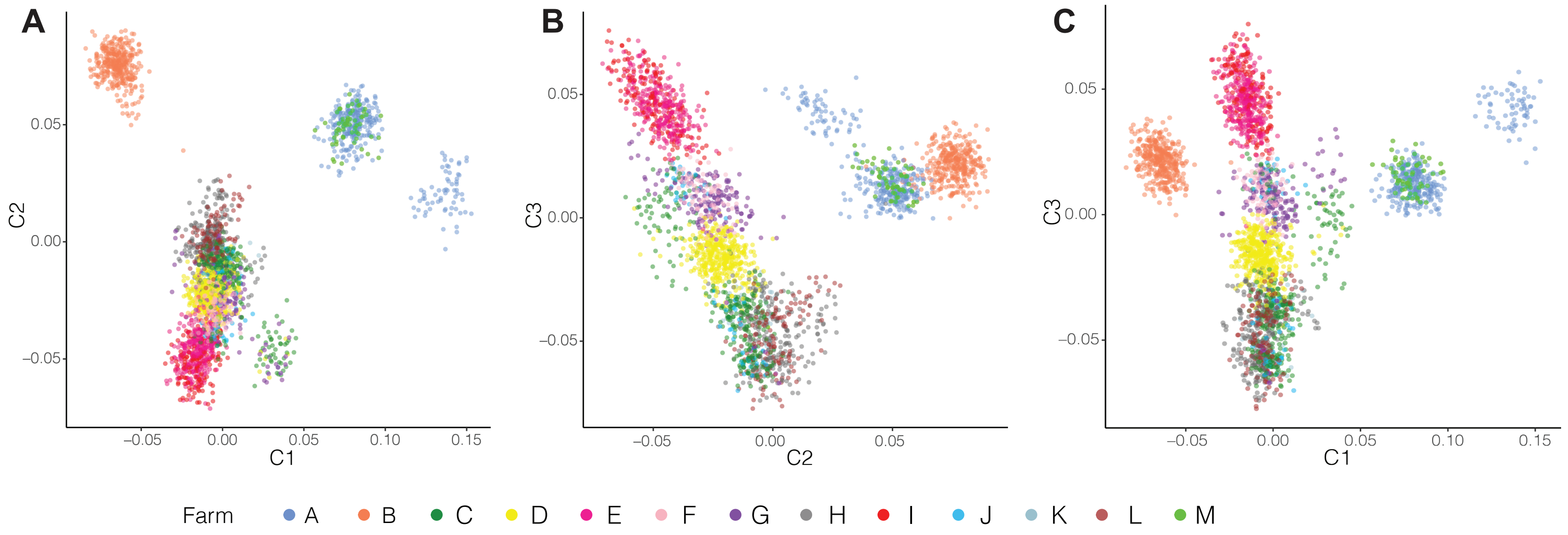

Figure S4. Multidimensional scaling (MDS) plots showing the first three dimensions (C1, C2, and C3) of the population structure for genotyped animals based on pairwise identity-by-state distance. Each point represents a genotyped animal and the colors of the points represent the origin farms for animals. **(A)** The MDS plot showing in the C1 and C2 dimensions. **(B)** The MDS plot showing in the C2 and C3 dimensions. **(C)** The MDS plot showing in the C1 and C3 dimensions.

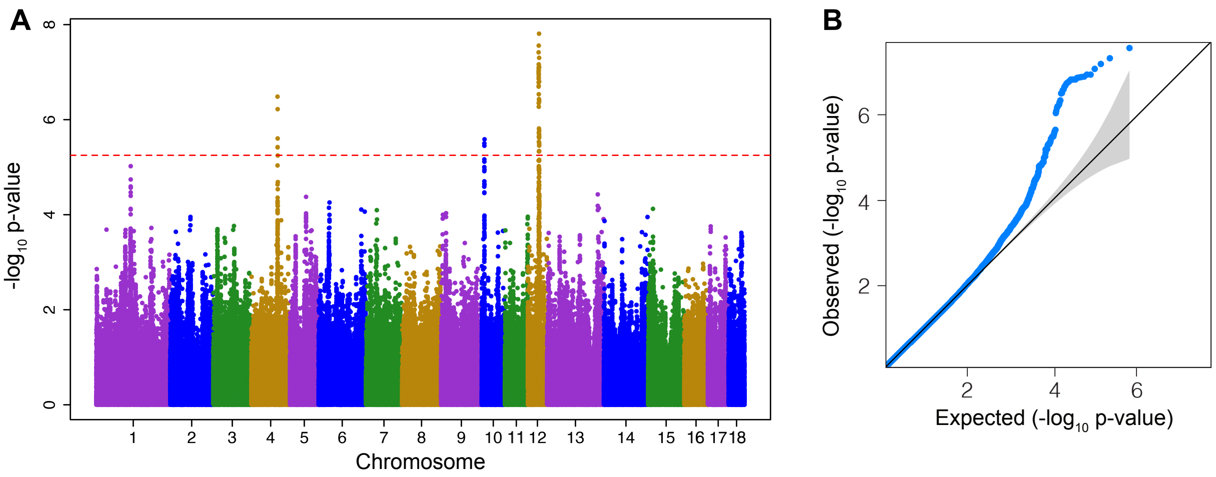

Figure S5. **(A)** Manhattan plot for the eosinophil concentration in Blood 3 (EOSB3) from univariate SSGWAS, significant SNPs were determined with the genome-wise false discovery rate at 0.05 (red dashed line). **(B)** Quantile-Quantile plot for EOSB3, the grey region represents a 95% confidence interval. Genomic inflation factor (λ) = 1.01.

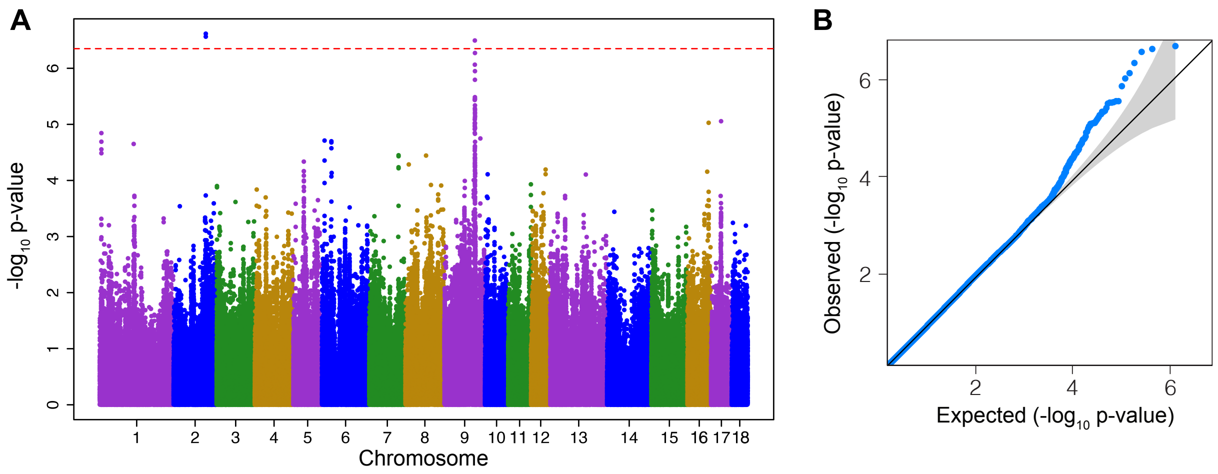

Figure S6. **(A)** Manhattan plot for the change of monocyte concentration from Blood 1 to Blood 3 (MONOΔ13) from univariate SSGWAS, significant SNPs were determined with the genome-wise false discovery rate at 0.05 (red dashed line). **(B)** Quantile-Quantile plot for MONOΔ13, the grey region represents a 95% confidence interval. Genomic inflation factor (λ) = 0.98.

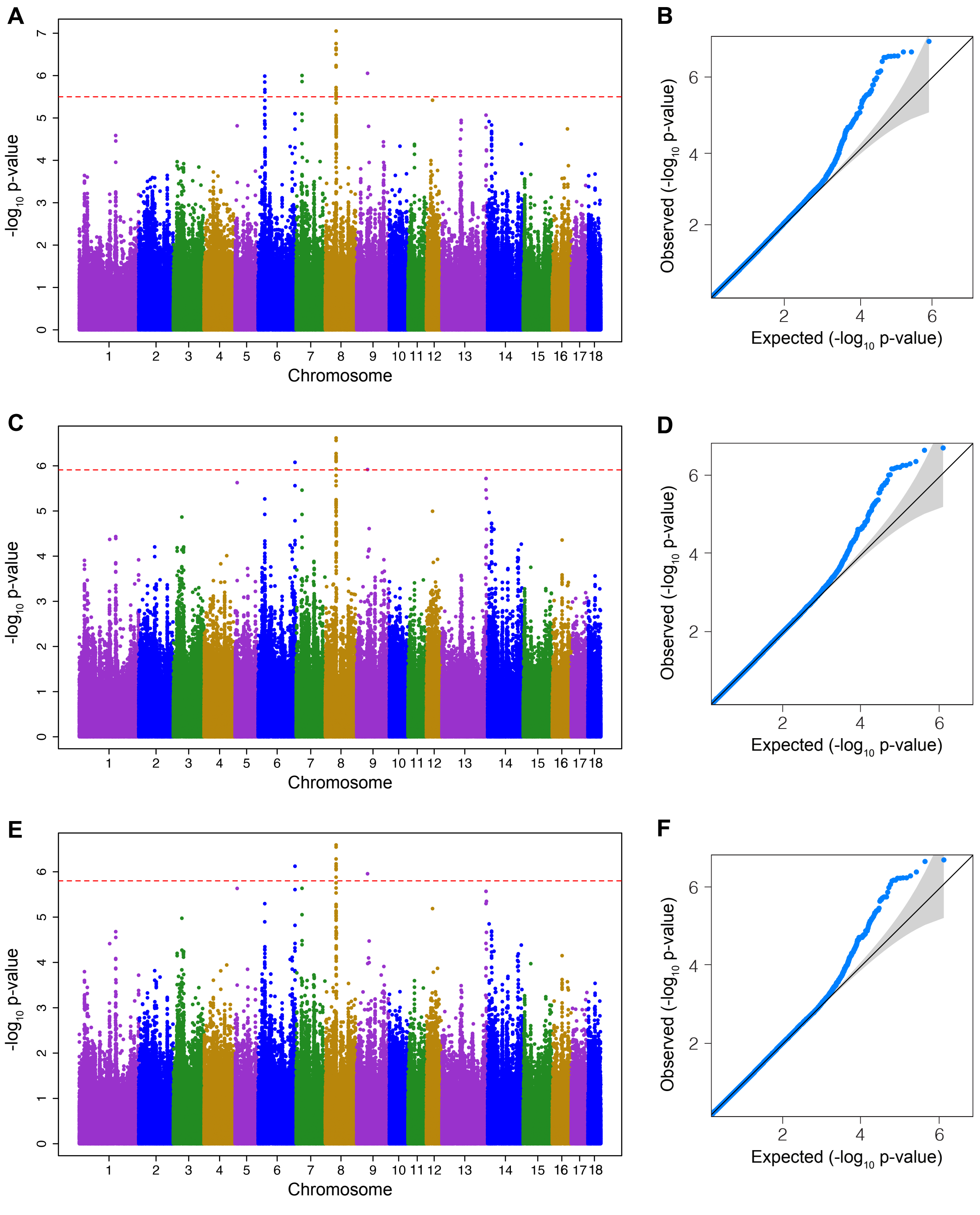

Figure S7. Manhattan plots (**A, C, E**) and Quantile-Quantile plots (**B, D, F**) for the mean corpuscular hemoglobin (MCH) in Blood 1, Blood 3, and Blood 4 from multivariate SSGWAS, respectively. Significant SNPs were determined with the genome-wise false discovery rate at 0.05 (the red dashed line in the Manhattan plot). The grey region in the Quantile-Quantile plot represents a 95% confidence interval. Genomic inflation factors (λ) were 1.01, 0.99, and 1 for MCH in Blood 1, Blood 3, and Blood 4, respectively.

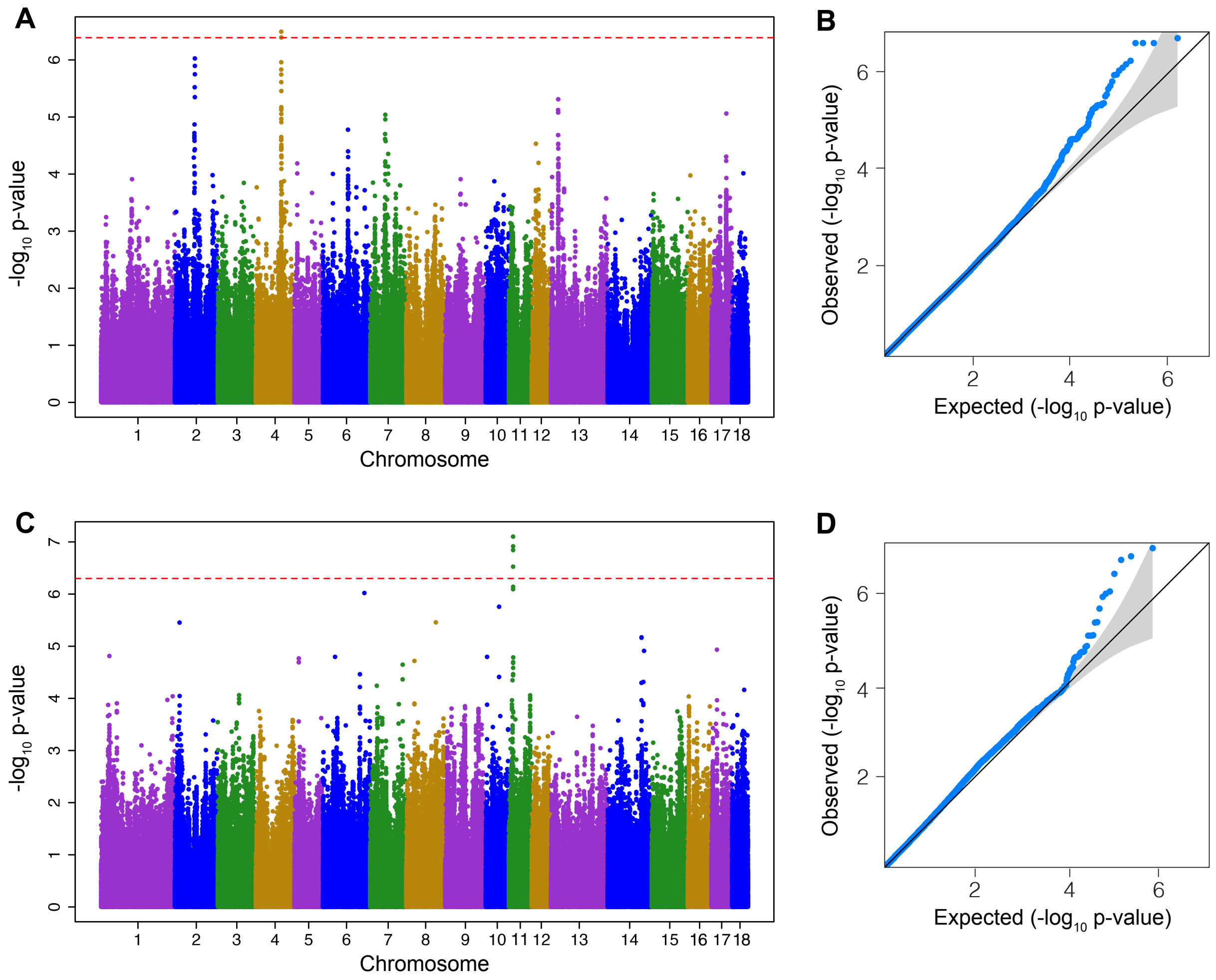

Figure S8. Manhattan plots (**A, C**) and Quantile-Quantile plots (**B, D**) for the mean corpuscular hemoglobin concentration (MCHC) in Blood 3 and for the change of MCHC from Blood 1 to Blood 4 (Δ14) from univariate SSGWAS, respectively. Significant SNPs were determined with the genome-wise false discovery rate at 0.05 (the red dashed line in the Manhattan plot). The grey region represents a 95% confidence interval in the Quantile-Quantile plot. Genomic inflation factors (λ) were 1 for MCHC in Blood 3 and 1.06 for MCHC in Δ14.

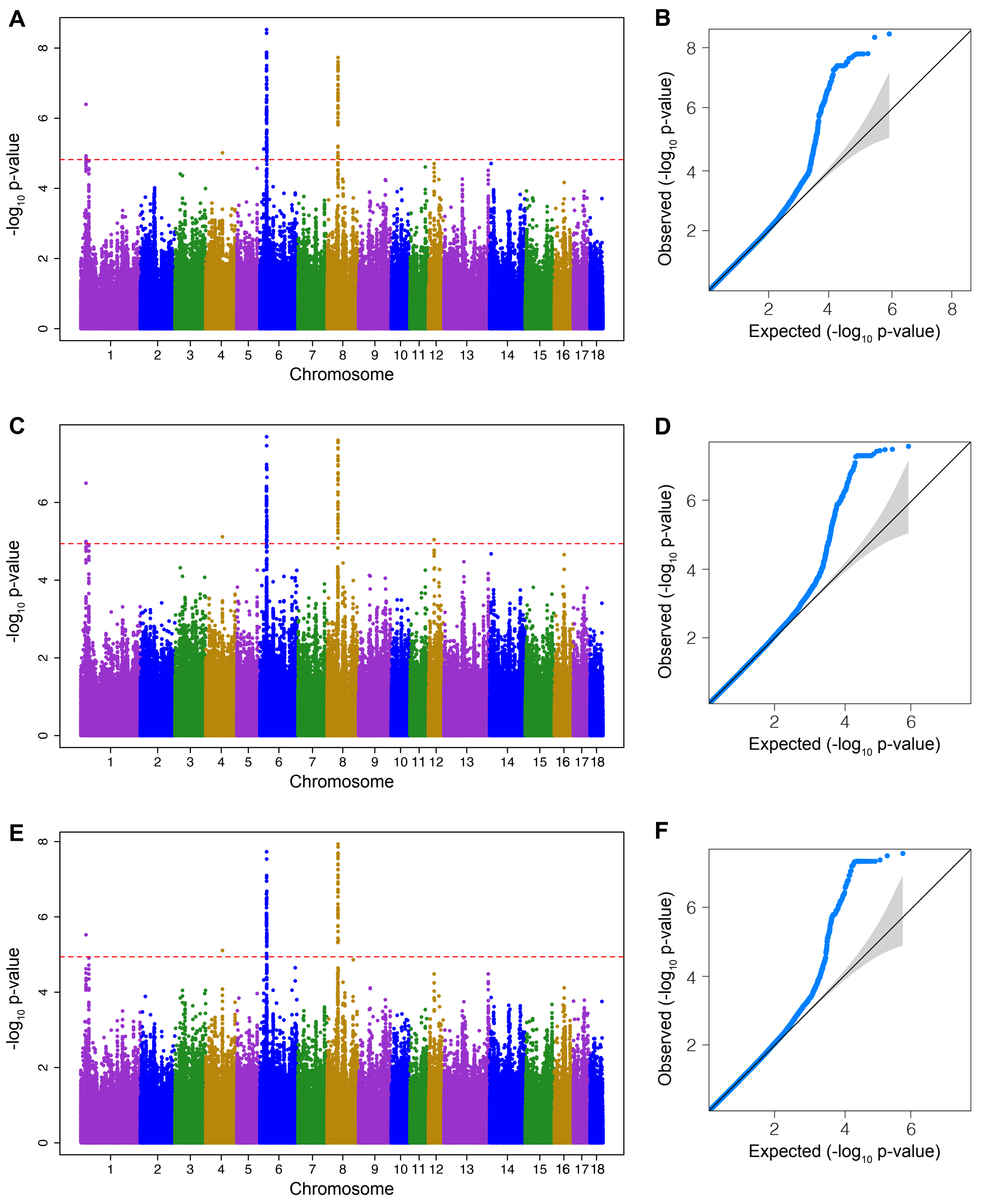

Figure S9. Manhattan plots (**A, C, E**) and Quantile-Quantile plots (**B, D, F**) for the mean corpuscular volume (MCV) in Blood 1, Blood 3, and Blood 4 from multivariate SSGWAS, respectively. Significant SNPs were determined with the genome-wise false discovery rate at 0.05 (the red dashed line in the Manhattan plot). The grey region represents a 95% confidence interval in the Quantile-Quantile plot. Genomic inflation factors (λ) were 0.99, 1, and 0.99 for MCV in Blood 1, Blood 3, and Blood 4, respectively.

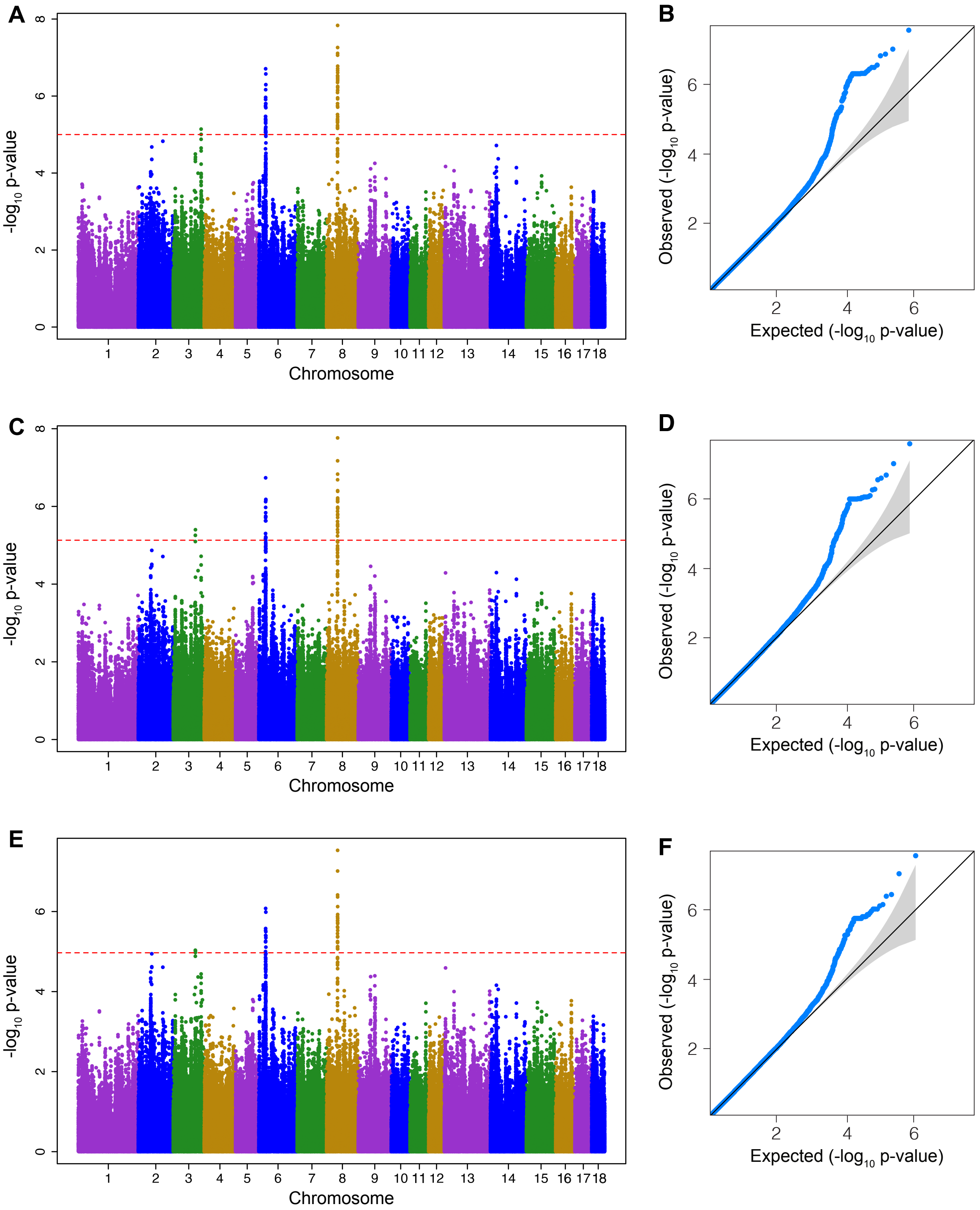

Figure S10. Manhattan plots (**A, C, E**) and Quantile-Quantile plots (**B, D, F**) for the red blood cell concentration (RBC) in Blood 1, Blood 3, and Blood 4, respectively. Significant SNPs were determined with the genome-wise false discovery rate at 0.05 (the red dashed line in the Manhattan plot). The grey region represents a 95% confidence interval in the Quantile-Quantile plot. Genomic inflation factors (λ) were 1, 1, and 0.99 for RBC in Blood 1, Blood 3, and Blood 4, respectively.

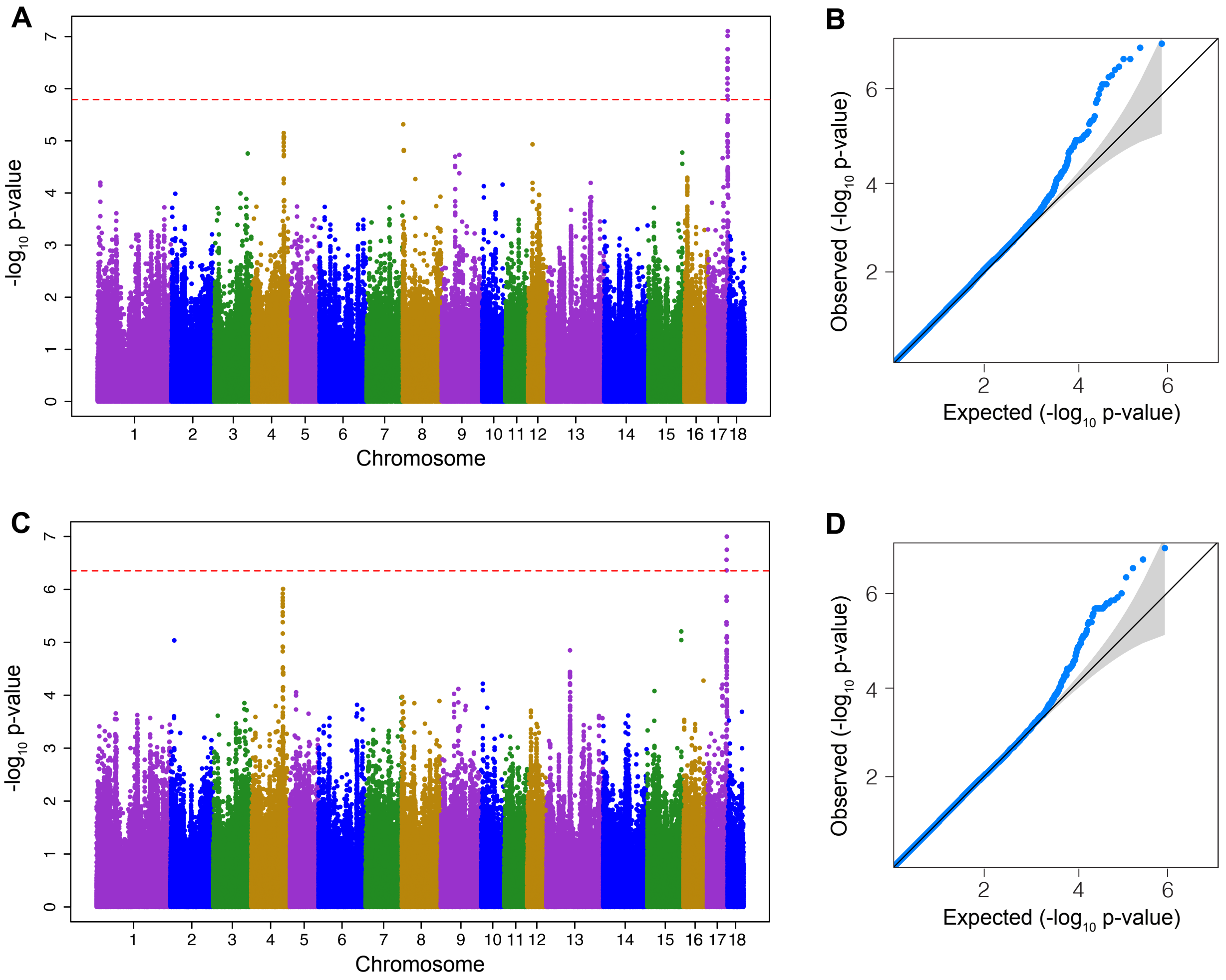

Figure S11. Manhattan plots (**A, C**) and Quantile-Quantile plots (**B, D**) for the mean platelet volume (MPV) in Blood 1 and Blood 4 from multivariate SSGWAS, respectively. Significant SNPs were determined with the genome-wise false discovery rate at 0.05 (the red dashed line in the Manhattan plot). The grey region represents a 95% confidence interval in the Quantile-Quantile plot. Genomic inflation factors (λ) were 1.02 and 1.01 for MPV in Blood 1 and Blood 4, respectively.

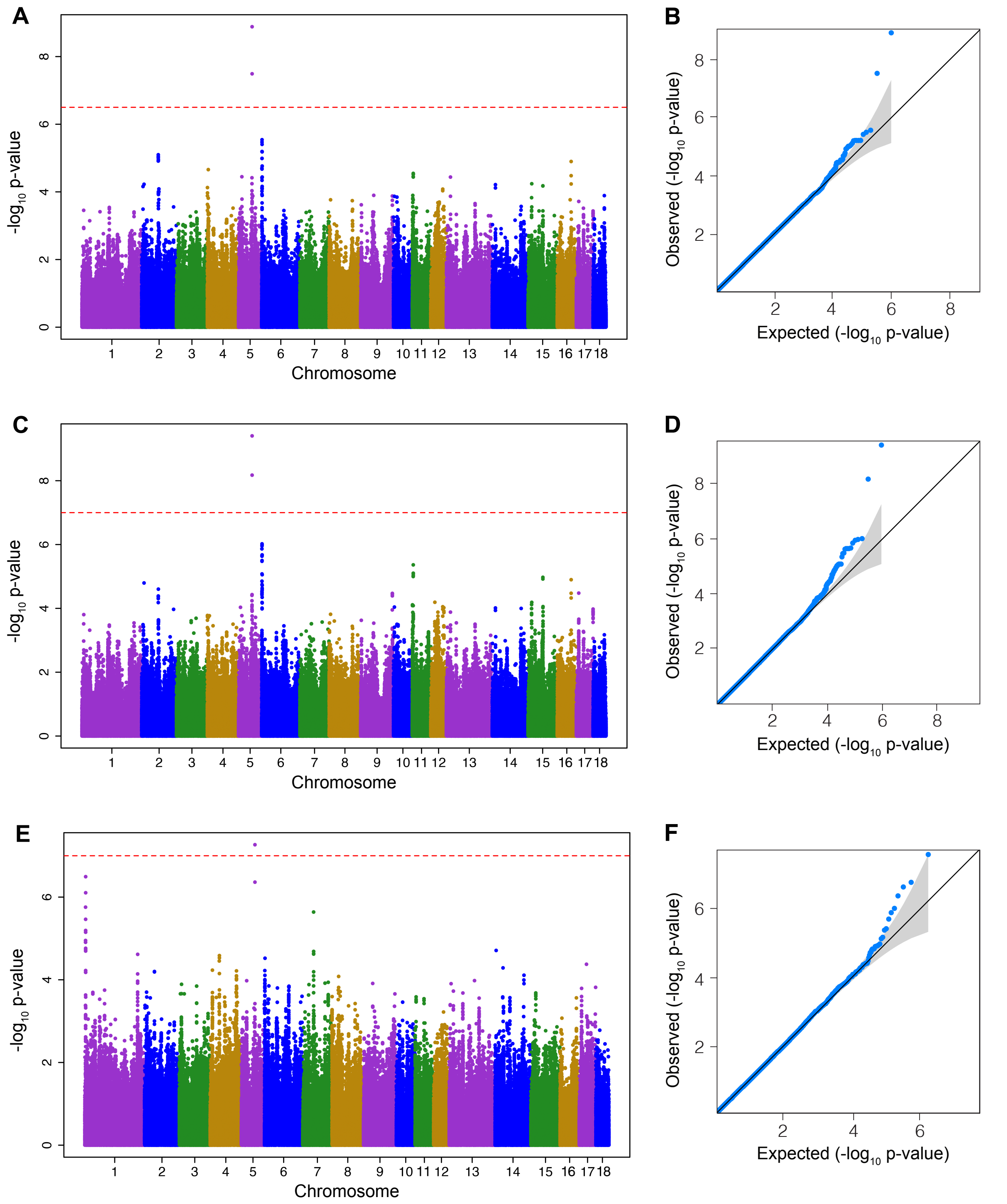

Figure S12. Manhattan plots (**A, C, E**) and Quantile-Quantile plots (**B, D, F**) for the platelet concentration (PLT) in Blood 1, Blood 3, and Blood 4 from multivariate SSGWAS, respectively. Significant SNPs were determined with the genome-wise false discovery rate at 0.05 (the red dashed line in the Manhattan plot). The grey region represents a 95% confidence interval in the Quantile-Quantile plot. Genomic inflation factors (λ) were 0.99, 1, and 1 for PLT in Blood 1, Blood 3, and Blood 4.
