# Supplementary material for: Investigating the genetic architecture of disease resilience in pigs by genome-wide association studies of complete blood count traits collected from a natural disease challenge model
Source: BMC Genomics. 2021 Jul 13;22:535. doi: 10.1186/s12864-021-07835-4 (PMC8278769; doi:10.1186/s12864-021-07835-4)
Supplement: Supplementary file 3 — Additional file 3: Table S2. Browsing regions for candidate genes located within 1-Mb on either side of lead SNPs (FDR < 0.10) associated with complete blood cell count traits; Figure S13. Haplotype block pattern (r2-scheme) for the region of candidate genes on SSC2 located within the maximum distance of 1 Mb on either side of the top lead SNP4 (SSC2, 120,341,201 bp); Figure S14. Haplotype block pattern (r2-scheme) for the region of candidate genes on SSC4 located within the maximum distance of 1 Mb on either side of the top lead SNP6 (SSC4, 91,591,493 bp); Figure S15. Haplotype block pattern (r2-scheme) for the region of candidate genes on SSC6 located within the maximum distance of 1 Mb on either side of the top lead SNP7 (SSC6, 28,511,423 bp); Figure S16. Haplotype block pattern (r2-scheme) for SNPs (40,946,144 bp to 41,198,574 bp) on SSC8 located within the maximum distance of 1 Mb on either side of the top lead SNP8 (SSC8, 41,156,538 bp). [file 12864_2021_7835_MOESM3_ESM.pdf]

Table S2. Browsing regions for candidate genes located within 1-Mb on either side of lead SNPs (*FDR* < *0*.10) associated with complete blood count traits.

Traits[1]	Blood[2]	Browsing region<br>*Sus scrofa* chromosome (SSC): position (bp)
EOS	Blood 3	SSC1: 110,498,112bp – 112,498,112bp; SSC4: 92,647,202bp – 94,647,202bp; SSC10: 7,186,695bp – 9,186,695bp; SSC12: 35,308,994bp – 37,308,994bp.
MONO	Δ13	SSC2: 119,341,201bp – 121,341,201bp; SSC9: 104,461,701bp – 106,461,701bp
MCH	Blood 1, 3, 4	SSC5: 9,683,166bp – 11,683,166bp; SSC6: 27,511,423bp – 29,511,423bp; SSC6: 164,588,523bp – 166,588,523bp; SSC7: 22,056,369bp – 24,056,369bp; SSC8: 40,156,538bp – 42,156,538bp; SSC9: 39,919,049bp – 41,919,049bp; SSC12: 23,001,577bp – 25,001,577bp; SSC13: 199,855,463bp – 201855463bp.
MCV	Blood 1, 3, 4	SSC1: 17,792,764bp – 19,792,764bp; SSC4: 76,486,634bp – 78,486,634bp; SSC6: 27,511,423bp – 29,511,423bp; SSC8: 40,156,538bp – 42,156,538bp; SSC12: 21,234,265bp – 23,234,265bp.
RBC	Blood 1, 3, 4	SSC2: 59,174,089bp – 61,174,089bp; SSC2: 104,736,448bp – 124,736,448bp; SSC3: 96,212,688bp – 98,212,688bp; SSC3: 120,086,804bp – 122,086,804bp; SSC6: 27,511,423bp – 29,511,423bp; SSC8: 40,156,538bp – 42,156,538bp.
MPV	Blood 1, 4	SSC4: 110,541,124bp – 112,541,124bp; SSC17: 58,739,745bp – 60,739,745bp.
PLT	Blood 1, 3, 4	SSC1: 1,655,014bp – 3,655,014bp; SSC5: 63,520,638bp – 65,520,638bp.

[1]The category of traits that associated with candidate genes. EOS: eosinophil concentration; MONO: monocyte concentration; MCV: mean corpuscular volume; RBC: red blood cell concentration; MCH: mean corpuscular hemoglobin; MPV: mean platelet volume; PLT: platelet concentration.

[2] Blood 1, Blood 3, and Blood4: CBC measured in blood samples collected at 2-weeks before, and 2- and 6-weeks after a polymicrobial infectious challenge; Δ13: the change of CBC measures from Blood 1 collected at 2-weeks before the challenge to Blood 3 collected at 2-weeks after the challenge; Δ14: the change of CBC measures from Blood 1 collected at 2-weeks before the challenge to Blood 4 collected at 6-weeks after the challenge.

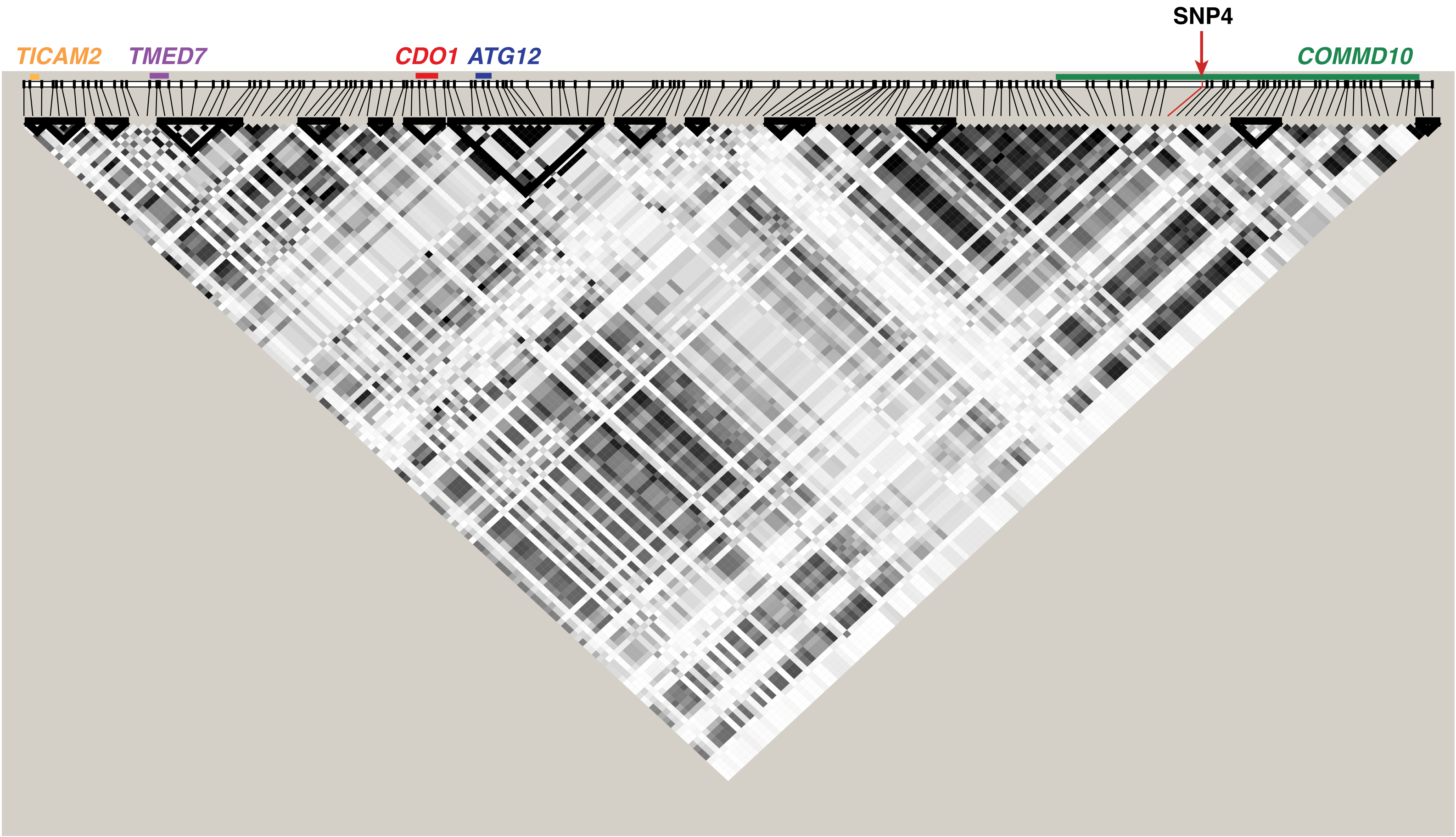

Figure S13. Haplotype block pattern (r^2-scheme) for the region of candidate genes on SSC2 located within the maximum distance of 1-Mb on either side of the top lead SNP4 (SSC2, 120,341,201bp, the most significant SNP at genome-wise false discovery rate < 0.05 with a group of supportive SNPs in this region), based on the linkage disequilibrium (LD, measured as r^2) among the SNPs within this region. The SNPs in each triangle box are suggested to be grouped in one haplotype block based on LD information.

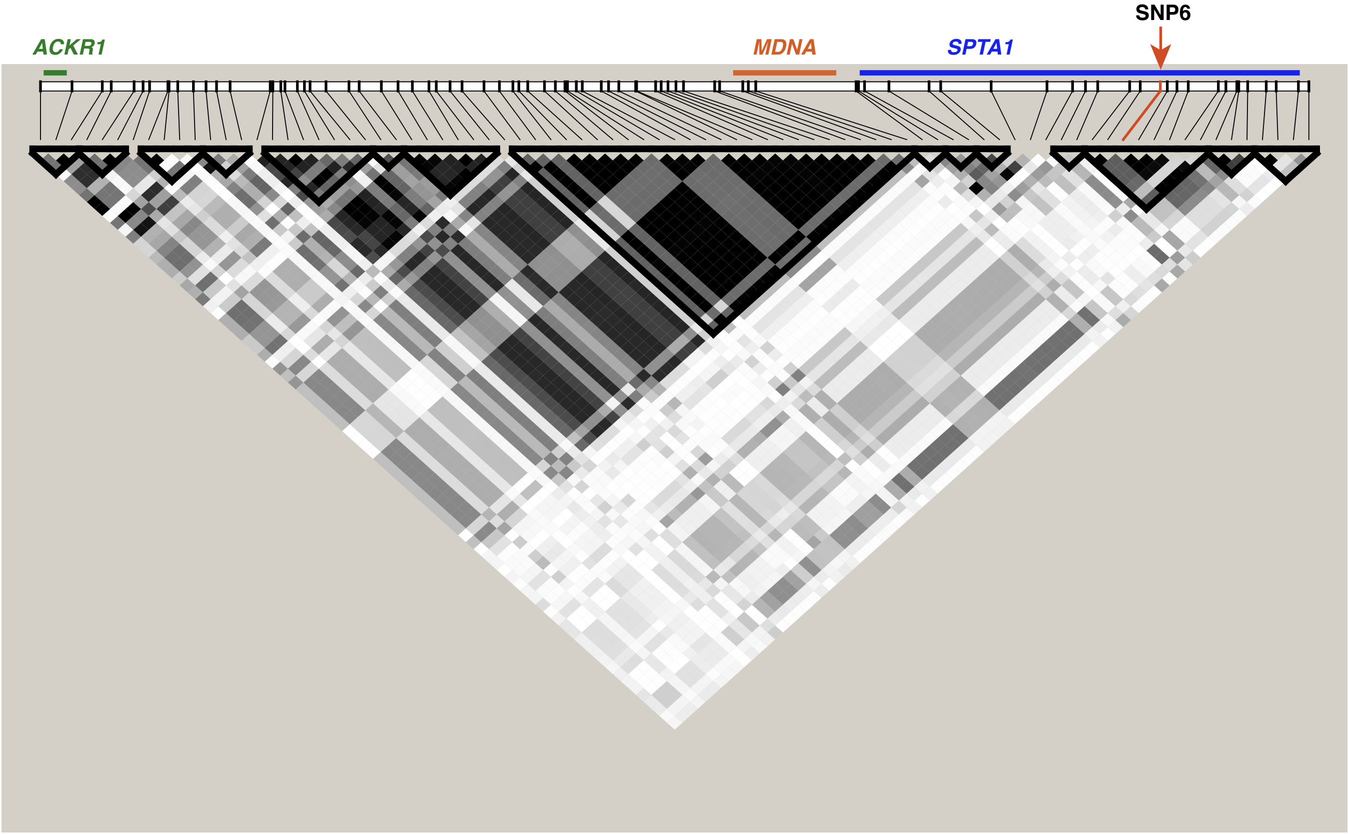

Figure S14. Haplotype block pattern (r^2-scheme) for the region of candidate genes on SSC4 located within the maximum distance of 1-Mb on either side of the top lead SNP6 (SSC4, 91,591,493bp, the most significant SNP at genome-wise false discovery rate < 0.05 with a group of supportive SNPs in this region), based on the linkage disequilibrium (LD, measured as r^2) among the SNPs within this region. The SNPs in each triangle box are suggested to be grouped in one haplotype block based on LD information.

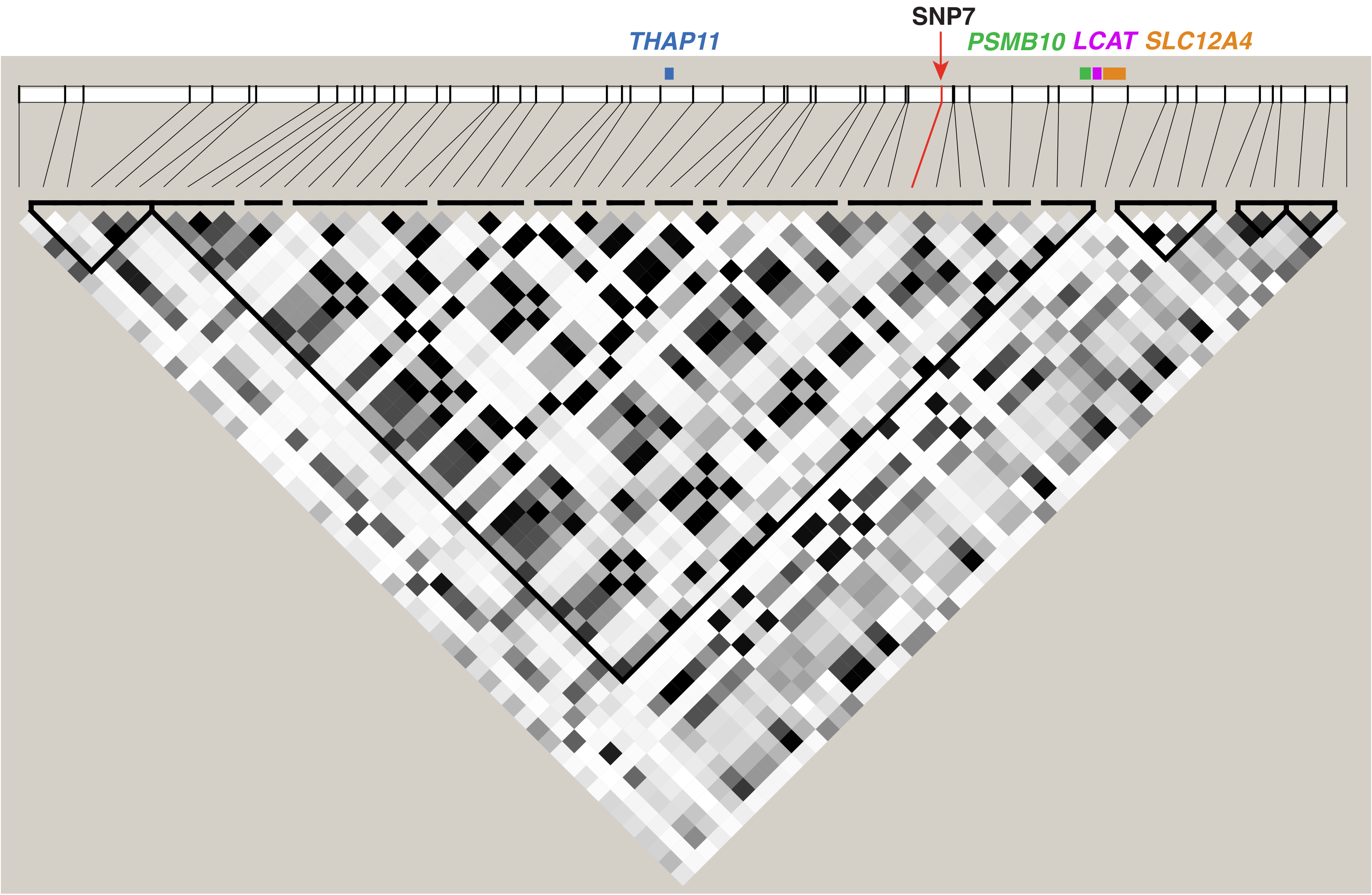

Figure S15. Haplotype block pattern (r^2-scheme) for the region of candidate genes on SSC6 located within the maximum distance of 1-Mb on either side of the top lead SNP7 (SSC6, 28,511,423bp, the most significant SNP at genome-wise false discovery rate < 0.05 with a group of supportive SNPs in this region), based on the linkage disequilibrium (LD, measured as r^2) among the SNPs within this region. The SNPs in each triangle box are suggested to be grouped in one haplotype block based on LD information.

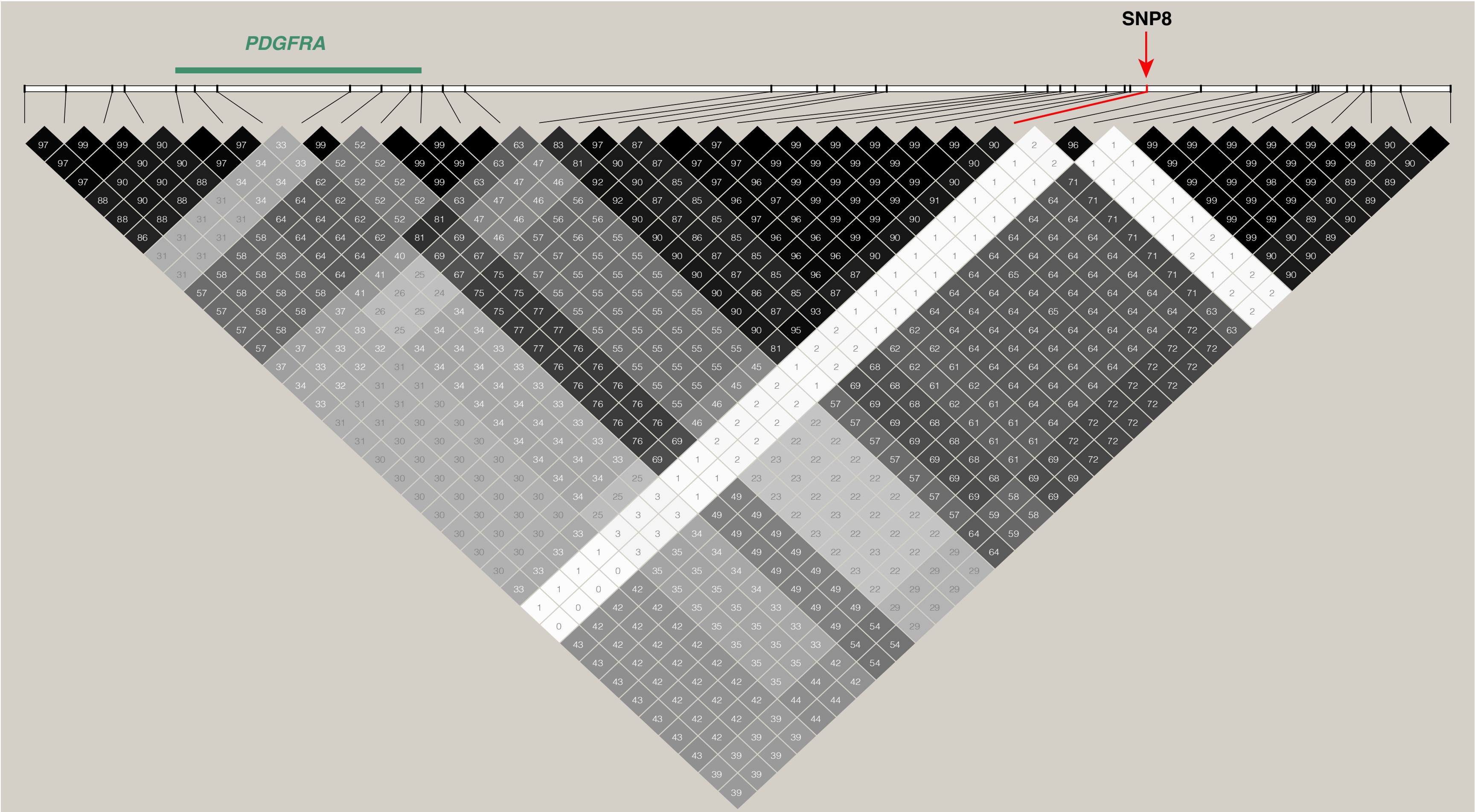

Figure S16. Haplotype block pattern (r^2-scheme) for SNPs (40,946,144bp to 41,198,574bp) on SSC8 located within the maximum distance of 1-Mb on either side of the top lead SNP8 (SSC8, 41,156,538bp, the most significant SNP at genome-wise false discovery rate < 0.05 with a group of supportive SNPs in this region), based on the linkage disequilibrium (LD, measured as r^2) among the SNPs within this region.
